# Electrospun Scaffolds Functionalized with a Hydrogen Sulfide Donor Stimulate Angiogenesis

**DOI:** 10.1021/acsami.2c06686

**Published:** 2022-06-17

**Authors:** Tianyu Yao, Teun van Nunen, Rebeca Rivero, Chadwick Powell, Ryan Carrazzone, Lilian Kessels, Paul Andrew Wieringa, Shahzad Hafeez, Tim G.A.M. Wolfs, Lorenzo Moroni, John B. Matson, Matthew B. Baker

**Affiliations:** † Complex Tissue Regeneration, MERLN Institute for Technology-Inspired Regenerative Medicine, Maastricht University, Universiteitssingel 40, Maastricht 6229 ER, The Netherlands; ‡ Shaanxi Key Laboratory of Degradable Biomedical Materials and Shaanxi R&D Center of Biomaterials and Fermentation Engineering, School of Chemical Engineering, Northwest University, Taibai North Road 229, Xi’an, Shaanxi, 710069, China; § Chemistry Department, Macromolecules Innovation Institute, Virginia Tech, 1075 Life Science Circle, Blacksburg, Virginia 24061, United States; ∥ Department of Pediatrics, Universiteitssingel 50, Maastricht University, Maastricht 6229 ER, The Netherlands

**Keywords:** electrospun, *N*-thiocarboxyanhydrides, click functionalization, angiogenesis, reactive sulfur species

## Abstract

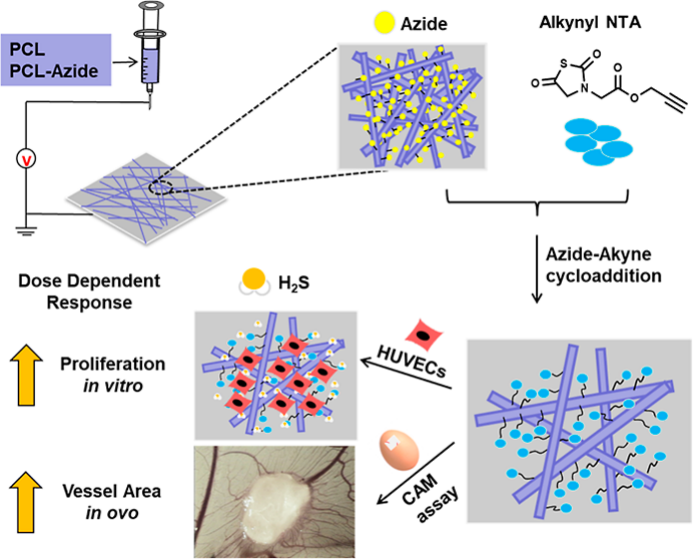

Tissue-engineered constructs are currently limited by the lack of vascularization necessary for the survival and integration of implanted tissues. Hydrogen sulfide (H_2_S), an endogenous signaling gas (gasotransmitter), has been recently reported as a promising alternative to growth factors to mediate and promote angiogenesis in low concentrations. Yet, sustained delivery of H_2_S remains a challenge. Herein, we have developed angiogenic scaffolds by covalent attachment of an H_2_S donor to a polycaprolactone (PCL) electrospun scaffold. These scaffolds were engineered to include azide functional groups (on 1, 5, or 10% of the PCL end groups) and were modified using a straightforward click reaction with an alkyne-functionalized *N*-thiocarboxyanhydride (alkynyl-NTA). This created H_2_S-releasing scaffolds that rely on NTA ring-opening in water followed by conversion of released carbonyl sulfide into H_2_S. These functionalized scaffolds showed dose-dependent release of H_2_S based on the amount of NTA functionality within the scaffold. The NTA-functionalized fibrous scaffolds supported human umbilical vein endothelial cell (HUVEC) proliferation, formed more confluent endothelial monolayers, and facilitated the formation of tight cell–cell junctions to a greater extent than unfunctionalized scaffolds. Covalent conjugation of H_2_S donors to scaffolds not only promotes HUVEC proliferation *in vitro*, but also increases neovascularization *in ovo*, as observed in the chick chorioallantoic membrane assay. NTA-functionalized scaffolds provide localized control over vascularization through the sustained delivery of a powerful endogenous angiogenic agent, which should be further explored to promote angiogenesis in tissue engineering.

## Introduction

Controlling angiogenesis to promote vascularization of engineered tissue remains a challenge in the upscaling and translation of tissue regeneration strategies. A careful design of both the physical cellular environment (scaffold or matrix) and the spatiotemporal signaling of cells with chemical cues is necessary for a successful approach. Traditional approaches consist of utilizing naturally sourced materials and growth factors to increase vascularization within tissue-engineered constructs; however, these strategies are often limited by control over properties, batch-to-batch differences (*e.g.*, matrigel, fibrin), and powerful but fragile and expensive vascular endothelial growth factor (VEGF), which can have significant off-target effects. Ideally, control of angiogenesis could be accomplished *via* a fully synthetic system, where both the scaffold and signaling components are synthetically accessible and scalable. The cell’s native extracellular matrix (ECM) provides both structural support (physical cues) and biological communication (chemical cues) to guide tissue formation.^1−3^ Synthetic ECMs recreating this nanofibrous network have shown a significant promise,^4,5^ but recapitulating and controlling bioactive signals remains a challenge.

As a method to recapitulate the fibrous nature of native ECM,^6−8^ electrospinning has emerged as a simple, cost-effective, and versatile material-processing technique that is used to fabricate continuous, ultrafine fibers from the micro- to nanoscale.^9−11^*Via* control of the electrospinning parameters (*e.g.*, voltage, flow rate, and working distance), one can straightforwardly control the morphologies, diameters, and pore sizes of nanofibers.^9,12^ The large specific surface area, high porosity, and spatial interconnectivity of electrospun nanofibers favor endothelial cell adhesion, proliferation, migration, and angiogenesis.^13−15^ Niu and Galluzzi fabricated a tubular nanofibrous scaffold based on collagen and hyaluronic acid, which was reported to support endothelial cell proliferation, phenotypic shape, and endothelialization.^16^ Moreover, electrospun scaffolds can be further functionalized by incorporation of or conjugation with angiogenic components (*e.g.*, growth factors^17−19^ and other bioactive molecules^20,21^) to better control the angiogenesis of endothelial cells on the scaffolds. Del Gaudio *et al.* fabricated a VEGF-loaded gelatin nanofibrous scaffold.^17^ Both the *in vitro* and *in vivo* studies showed that the VEGF-loaded scaffold induced angiogenic potential by promoting vessel formation. Previously, we have investigated endothelial cell behavior using electrospun scaffolds with geometrical cues and co-culture between human vascular endothelial cells (HUVECs) and hMSCs,^22,23^ but we envisioned that the inclusion of chemical cues to promote angiogenesis could further enhance the capabilities of such electrospun scaffolds.

Several methods exist to incorporate angiogenic chemical cues into synthetic ECM materials, such as the incorporation of specific peptide sequences or sustained release of growth factors. Much less explored is the delivery of gasotransmitters, which are powerful endogenous signaling molecules (*e.g.*, nitric oxide). The most recently discovered gasotransmitter, hydrogen sulfide (H_2_S), has been recognized as an important physiological and pathological signaling molecule that mediates and promotes angiogenesis.^24,25^ In endothelial cells, H_2_S is generated from cysteine by enzymes such as cystathionine β-synthase (CBS), cystathionine γ-lyase (CSE), and the combined action of cysteine aminotransferase (CAT) and 3-mercaptopyruvate sulfurtransferase (3-MST).^26−29^ H_2_S plays a role in angiogenesis by promoting endothelial cell proliferation^30,31^ and stimulating angiogenesis *in vitro* and *in vivo*. For example, the addition of exogenous H_2_S stimulated capillary morphogenesis of HUVECs on Matrigel,^30^ resulted in a concentration-dependent increase in the vessel length in the chicken chorioallantoic membrane (CAM) assay,^30^ and increased the collateral vessel growth, capillary density, and regional tissue blood flow in the ischemic hind limb muscles in mice.^32^ Overall, this novel gasotransmitter has shown powerful early results in the promotion of angiogenesis.

Because H_2_S is a gas, its delivery in biological studies is usually accomplished using small molecules that react *in vitro* or *in vivo* to generate H_2_S, termed H_2_S donors. The most widely and easily used class of H_2_S donors in biological studies are the sulfide salts, sodium hydrosulfide (NaSH), and sodium sulfide (Na_2_S),^33−35^ yet upon dissolution in an aqueous solution, these salts generate a large amount of H_2_S over a short time period,^36,37^ far from the slow and continuous H_2_S generation *in vivo.*(38) Engineered small-molecule H_2_S donors can enable sustained H_2_S release, which can more effectively regulate endothelial cell behavior.^37,39^ Despite the improvements that organic H_2_S donors provide over sulfide salts, small molecule donors can quickly diffuse from scaffolds, creating a need for H_2_S-releasing scaffolds and macromolecules^40^ with covalently attached H_2_S donors that enable sustained and controllable release rates. In 2016, we reported on the use of *N*-thiocarboxyanhydrides (NTAs) as H_2_S donors.^41^ NTAs undergo ring-opening, triggered by water or biological nucleophiles such as amines, to release carbonyl sulfide (COS), which is converted into H_2_S by the ubiquitous enzyme carbonic anhydrase (CA).^42^ More recently, we showed that useful functional groups such as alkynes could be installed onto NTAs to enable conjugation to other constructs, such as polymers and proteins.^43^

Motivated by the simplicity of electrospinning for the fabrication of ECM-mimicking fibrous scaffolds, combined with the ability of NTAs to release H_2_S, we sought to prepare a straightforward and simple tissue engineering scaffold to stimulate angiogenesis. Previous work has shown promise for the generation of H_2_S-releasing electrospun scaffolds;^44^ however, their ability to promote angiogenesis has never been explored. In our approach, we envisioned the preparation of “clickable” PCL scaffolds *via* the modular mixing of small amounts of a functional, low-molecular-weight azide-terminated PCL with a high-molecular-weight PCL. This strategy should result in a controllable density of azides on the scaffold,^45^ which after a postfabrication functionalization step, could provide surface-bound, H_2_S-releasing NTAs. We envisioned that this modular approach would allow us to measure the effects of NTA loading on the endothelial cell behavior *in vitro*, with HUVECs, and *in ovo*, in a CAM assay. Taken together, we hypothesized that this simple approach could lead to scaffolds that effectively promote angiogenesis, without the need for growth factors, by mimicking both the structural and chemical signaling within the native ECM.

## Materials and Methods

### Synthesis

#### PCL–Azide Synthesis

In the first step, *p*-toluenesulfonyl-poly(ε-caprolactone) (PCL-OTs) was synthesized, which is an intermediate product to PCL azide. PCL-diol (3 g, 1.5 mmol) was dissolved in 5 mL of dry dichloromethane in a dried round bottom flask. *p*-Toluenesulfonyl chloride (0.69 g, 3.6 mmol, 2.4 equiv) and triethylamine (1.2 g, 12 mmol, 8 equiv) were dissolved in 7 mL of dichloromethane in a separate flask and added to the PCL-diol solution dropwise. After 24 h, the reaction mixture was filtered to remove insoluble particles. The filtrate was collected and washed with 0.1 M HCl, NaHCO_3_ (aq sat.), NaCl (aq sat.), and H_2_O, respectively. The organic phase was dried by rotary evaporation, and PCL-OTs was obtained (3.3 g, 95% yield). ^1^H NMR confirmed the formation of PCL-OTs with a high degree of tosylation (77%).

PCL-OTs (3.3 g, 1.4 mmol) was dissolved in 5 mL of anhydrous dimethylformamide (DMF) under dry nitrogen and in a dry flask. Sodium azide (NaN_3_) (0.55 g, 8 mmol, 6 equiv) was added to the reaction flask, and the reaction was allowed to stir for 24 h at 50 °C under dry nitrogen. Next, the reaction mixture was dried *via* rotary evaporation until minimal DMF was left, the crude was precipitated in cold water, centrifuged at 6000 relative centrifugal force, and the supernatant decanted to isolate the crude product. The product was taken up in CHCl_3_ and washed with H_2_O to remove traces of NaN_3_ and to obtain the product PCL-N_3_ after solvent removal and drying (2.6 g, 81% yield).

Shown in Figure S1 are the NMRs of the starting materials and intermediates. Peaks corresponding to the tosylate (Figure S1b,i,g,h) are observed at 2.46 ppm (alpha methylene) and 7.35–7.79 ppm (aromatic protons). Figure S1a shows the ^1^H NMR spectrum of the final product with a clear triplet of H (a”) proton (δ = 3.24 ppm) resulting from the alpha methylenes next to the azide end-groups. FTIR further confirmed the presence of the azide in the polymer with a strong absorbance band (16% absorbance) at 2095 cm^–1^ (Figure S3).

#### Alkynyl-NTA Synthesis

Alkynyl-NTA was synthesized according to a published 3-step procedure.^43^ In brief, 2-[(ethoxycarbonothioyl)thio]-acetic acid was added to iminodiacetic acid to generate iminodiacetic acid thiocarbamate (TCDA). TCDA was then monoesterified with propargyl alcohol. Finally, phosphorous tribromide was added to induce ring-closing and NTA formation, generating the desired product.

### Preparation of PCL-N_3_ Fibers and Characterization

A high-molecular-weight poly(ε-caprolactone) (PCL-80K) *M*_n_ ≈ 80,000 g/mol, and the lower-molecular-weight PCLs (PCL-2K and PCL-N_3_-2K) were mixed in different ratios (1, 5, and 10 wt % of PCL-N_3_ in final scaffold as described in Table S1) to give a final concentration of 15 wt % polymer in a CHCl_3_/DMF (4/1, v/v) solvent mixture. Note that while the amount of PCL-N_3_ was varied, the weight ratio between 80 and 2 kg/mol PCL was kept constant at 90:10 *via* the adjustment of the amount of nonfunctionalized PCL-2K. The polymer solution was stirred overnight to form a homogeneous solution for electrospinning. The electrospinning setup is a homemade machine for generating nanofibers, as reported before.^46^ Briefly, the polymer solution was loaded into a 5 mL syringe (BD biosciences) equipped with a stainless steel blunt-ended needle. The polymer solution was delivered to the needle *via* a silicon feed line. The flow rate was controlled by a syringe pump (Harvard Apparatus PHD 2000) at 1 mL/h. An aluminum plate was used as the collector connected to the ground. The distance between the tip and the collecting plate was set as 20 cm, and the electrospinning voltage was kept at 20 kV. The temperature was maintained at 25 °C, and the relative humidity maintained at 35%. The prepared fibrous scaffolds were dried overnight at room temperature to remove traces of solvent. The morphologies of PCL-N_3_ fibrous scaffolds were examined by a scanning electron microscopy (SEM; XL30; Philips). Fiber samples were coated with gold for 60 s before imaging. At least five areas were randomly selected to test the uniformity of the fibers. The diameters of electrospun fibers were quantified from SEM images by using Image J. For FTIR measurements, an ATR stage on a NICOLET iS50 FT-IR (Thermo Scientific) was used to measure the as-spun samples or, in the case of the neat PCL-N_3_, as a powder.

The electrospun fibers are referred to as 0% PCL-N_3_, 1% PCL-N_3_, 5% PCL-N_3_, and 10% PCL-N_3_ corresponding to 0, 1, 5, and 10 wt % PCL-N_3_ in the final polymer scaffolds, respectively.

### Click Modification of PCL-N_3_ with Alkyne MegaStokes

To optimize the azide–alkyne functionalization, different PCL-N_3_ scaffolds were treated with a click reaction solution, which contained 0.4 mM CuSO_4_·5H_2_O, 2 mM Na-Ascorbate, and 0.05 mM Alkyne MegaStokes dye in ethanol (10 mL) at room temperature. Sealed reaction vials were placed on an orbital shaker for a set time interval. Then, the fibrous scaffolds were washed with absolute ethanol three times and dried under nitrogen in the dark. The control was the 0% PCL-N_3_ fibrous scaffolds in the click reaction, and all the other processes were the same. In order to study the effect of time on the functionalization, the reaction was set with different time points (15 min; 30 min; 2 h and 4 h). Finally, samples were placed in a glass-bottom Petri dish (Ibidi) and imaged with a fluorescent microscope (Nikon Eclipse Ti–S).

### Click Conjugation of Alkynyl-NTA on PCL-N_3_ Electrospun Fibers

A click reaction between PCL-N_3_ and alkynyl-NTA was performed on different PCL-N_3_ fibrous scaffolds (1, 5, 10% azide). Each of the different electrospun scaffolds was placed in a glass reaction bottle with a 1:1 mixture of water and ethanol (2 mL) and NTA (2.13 mg, 10 μmol, in 500 μL of DMSO). Sodium ascorbate (0.3 mmol, 300 μL of freshly prepared 1 M solution in water) was added, followed by copper (II) sulfate pentahydrate (7.5 mg, 0.03 mmol, in 100 μL of water). The heterogeneous mixture was stirred vigorously overnight. Then, the scaffolds were washed for 15 min in an EDTA solution (0.01 M in Milli-Q water) followed by three washes, 30 min each, with absolute ethanol.

### Aminofluorescein Assay for NTA Presence

After the click reaction of the 10% PCL-N_3_ scaffolds with alkynyl-NTA, NTA-functionalized scaffolds were placed in a new glass reaction bottle wrapped in aluminum foil. A mixture of 6-aminofluorescein (0.1 mM) and triethylamine (0.2 mM) solutions in ethanol (10 mL) was added to the 10% PCL-N_3_/NTA scaffolds, and the reaction bottle was placed on the shaker for 20 min. Untreated 10% PCL-N_3_ scaffolds were placed in the same fluorescence solution serving as a control. Then, both control and functionalized samples were washed three times with ethanol and placed in a glass-bottom Petri dish (Ibidi). Finally, samples were imaged with a fluorescent microscope (Nikon Eclipse Ti–S).

### Methylene Blue Assay

After the click conjugation with alkynyl-NTA, 1.4 mg of each of the modified electrospun scaffolds was placed in a glass reaction bottle with 373 μL of 1X PBS buffer (pH 7.4), 2 μL of a solution of glycine (0.5 M in H_2_O), and 250 μL of a solution of CA (33.3 μM in 1X PBS). The bottles were placed on a shaker and allowed to react for 60 min.^43^ After this time, an aliquot of each sample (500 μL) was diluted with 500 μL of an FeCl_3_ solution (30 mM in 1.2 M HCl) and 500 μL of a dimethyl-*p*-phenylene diamine solution (20 mM in 7.2 M HCl). Each solution remained sealed in a glass vial for a minimum of 60 min before analysis.^47^ The absorbance for each aliquot was measured using a UV–vis spectrophotometer (Cary 60 UV–vis Spectrophotometer, 600–800 nm, 1 cm path length). For the kinetic runs, the same procedure was repeated with aliquots being taken out from the scaffold/glycine/CA solution at 5, 15, 30, 45, and 60 min.

### Cell Culture

HUVECs were obtained from Lonza and cultured according to the standard procedures of Lonza. Briefly, HUVECs were cultured in an endothelial growth medium (EGM, Lonza) containing EBM-2 basal medium (CC-3156) and EGM-2 SingleQuots supplements (CC-4176). Cultures were incubated under a humidified environment with 5% CO_2_ at 37 °C. The culture medium was changed every 2 days, and cultures were passaged at 80% confluence to prevent contact inhibition. Passages 4 through 8 were used in this study.

Electrospun scaffolds were punched into round pieces (15 mm) and washed in water 3 times. Prior to cell seeding, the scaffolds were sterilized with 70% ethanol for 30 min and dried in a biosafety cabinet. The sterilized scaffolds were placed on a 24-well plate and fixed by O-rings. After washing with sterilized water, scaffolds were incubated overnight in Matrigel (1:150 dilution in EGM) to aid protein adhesion and cell attachment. We also incubated scaffolds with FBS overnight to compare the effect of scaffolds after a different coating process. The treated scaffolds were seeded with a density of 2 × 10^4^ cells/cm^2^. The culture medium was refreshed every 2 days.

### Live/Dead Assay

Cell cytotoxicity was assessed using a live/dead assay kit (Invitrogen), which included two components, calcein AM and ethidium homodimer-1 (EthD-1), to simultaneously determine the existence of live and dead cells on electrospun scaffolds. Briefly, the scaffolds were washed with PBS, incubated in 1 μM calcein AM (staining live cells) and 6 μM EthD-1 (staining dead cells) in PBS for 30 min at 37 °C. The cells were then washed with PBS three times to remove excess dye. Finally, the samples were observed under a fluorescent microscope. As a result, live cells are stained green, and dead cells are red. The percentage of live cells was assessed by counting the number of calcein AM-stained viable cells and EthD-1-stained dead cells.

### Cell Viability and Proliferation

The cells grew and proliferated on the electrospun scaffolds for 1, 3, and 5 days. Cell viability was analyzed using a PrestoBlue assay according to the manufacturer’s protocol (Fisher Scientific). The PrestoBlue reagent (10% V/V) was mixed with EGM to prepare the PrestoBlue medium. 500 μL of the PrestoBlue medium was added to samples and then incubated at 37 °C for 30 min. 100 μL of media from the samples was transferred from each well into a black 96-well plate with a clear bottom. The fluorescence emission was measured at 540–570 nm excitation and 580–610 nm emission in a microplate reader (CLARIOstar, BMG LABTECH). The readout from the samples was corrected with a control (PrestoBlue medium).

The DNA content based on the total amount of DNA of each sample was quantitatively determined with the CyQUANT Cell Proliferation Assay Kit (Thermo Fisher Scientific) at 1, 3, and 5 days. The cells were first digested overnight with 250 μL Proteinase K in a Tris/EDTA solution at 56 °C. The CyQUANT GR dye and lysis buffer were prepared according to the manufacturer’s protocol. After freeze-thawing samples 3 times, 40 μL of the digested samples was transferred to a black 96-well plate, then lysed in 40 μL of the lysis buffer for 1 h at room temperature. The GR dye solution (80 μL) was added to each well. After incubating the samples at room temperature for 15 min, the fluorescence intensity of the samples was measured using a microplate reader.

### Immunostaining

The electrospun scaffolds with HUVECs were fixed with 4% formaldehyde for 30 min at room temperature. The samples were permeabilized with PBS containing 0.1% Triton-X 100 for 15 min. After washing with PBS, the samples were blocked with 5% goat serum in a 1% BSA/0.05% Tween-PBS solution for 1 h at room temperature to block nonspecific protein interactions. The samples were then incubated with the primary antibodies (CD31 and VE-Cadherin; 1:200 dilution in a blocking solution; Ki67/1:300 dilution in a blocking solution) overnight at 4 °C. After washing with a washing buffer (0.05% Tween 20 and 1% BSA in PBS), the secondary antibody (goat-anti mouse, Alexa Fluor 488, 1:200 dilution in a washing buffer) was incubated for 1 h at room temperature in the dark. In addition, the cell cytoskeleton was stained with a phalloidin solution for 1 h at room temperature. DAPI was used to stain the cell nucleus for 5 min and finally observed with fluorescence microscopy (Nikon Eclipse Ti–S).

### Chorioallantoic Membrane Assay

The chick embryo CAM assay was performed to assess the ability of NTA functionalized fibrous scaffolds to induce angiogenesis *in ovo*. Fertilized chicken eggs were purchased from Het Anker B.V., Netherlands. The eggs were incubated at 37 °C with approximately 50–55% relative humidity (9 eggs for each condition). On day 3, a window of 1 × 1.5 cm^2^ was gently opened with a rotary tool (Dremel) on the wide end of the egg without damaging the embryo. The shell and inner membrane were peeled off with sterile tweezers. About 1–1.5 mL of albumen was aspirated with a syringe in order to detach the developing membrane from the top part of the shell. The windows were closed with transparent tape to prevent dehydration and possible infections before putting the eggs back in the incubator. On incubation day 10, the sterilized scaffolds with a diameter of 4 mm were placed on the egg membrane between branches of the blood vessels. After 4 days of incubation, the scaffolds were imaged with the surrounding vessels under a Leica microscope. The quantification of vessel areas was processed by Image J using an automated script. Angiogenesis was evaluated by the area of the blood vessels around the scaffolds.

### Statistical Analysis

Statistical analysis was carried out using GraphPad Prism 8 software. All data are expressed as mean ± standard deviation. Data were statistically analyzed by one-way analysis of variance; column values were compared with the control values using the Holm-Sidak multiple-comparisons test. A probability value of less than 0.05 was considered significantly different. Levels of significance were as follows: *P ≤ 0.05, **P ≤ 0.005, ***P ≤ 0.0005, ****P ≤ 0.0001.

## Results and Discussion

### Fabrication of PCL-N_3_-Incorporated Electrospun PCL Fibers

In order to create scaffolds with a controlled NTA density, we sought to create an azide-functionalized electrospun scaffold that could be postfabrication functionalized with the alkynyl-NTA. We envisioned that the simple mixing of a low molecular weight functional polymer with a higher-molecular-weight polymer for mechanical integrity would allow the straightforward creation of functionalized fibers. According to a previous study, large amounts of the azide groups (80%) were presented on the surface of PCL fibers with a similar mixing approach, suggesting enrichment of the functional groups on the surface of the scaffold.^45^ This blending of small molecular weight PCL-N_3_ with a higher-molecular-weight PCL for electrospinning was considered an efficient way to control the density of functionality and fabricate fibers with a clickable surface.

A small-molecular-weight PCL-N_3_ was first synthesized in order to achieve this approach. A diazide-terminated PCL was synthesized from the commercially available PCL diol (2 kg/mol) *via* a tosylation/azidation pathway.^45^ These high-yielding reactions facilitated the straightforward production of PCL-N_3_ on a multigram scale.

**Figure 1:**
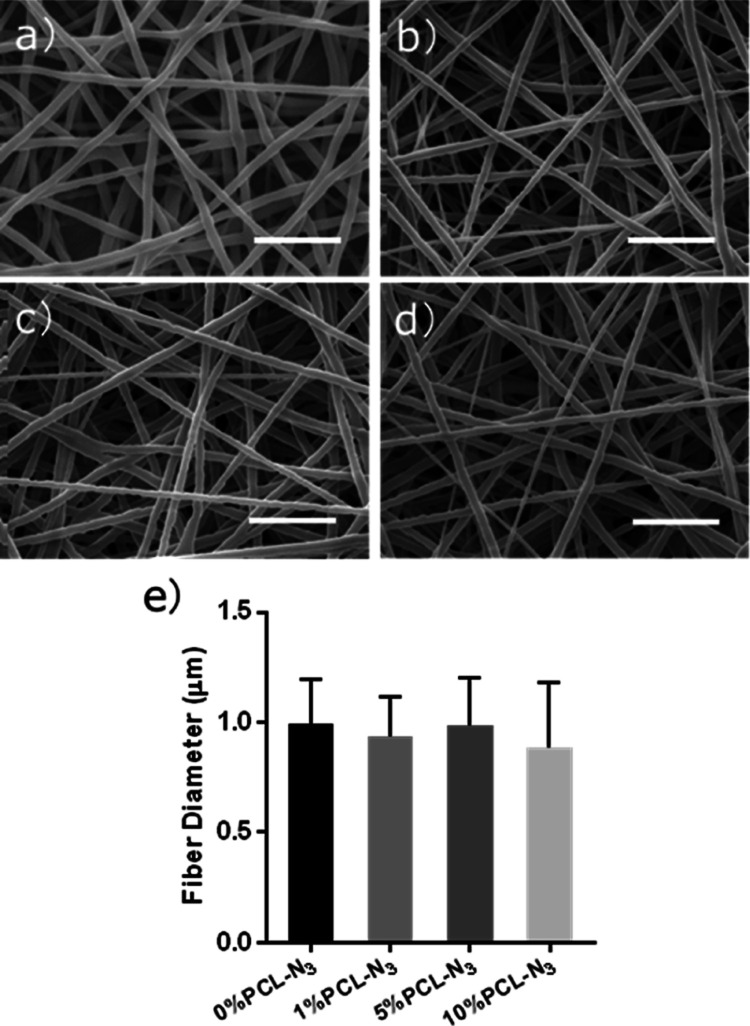
SEM images of the (a) 0% PCL-N_3_-electrospun scaffolds, (b) 1% PCL-N_3_-electrospun scaffolds, (c) 5% PCL-N_3_-electrospun scaffolds and (d) 10% PCL-N_3_-electrospun scaffolds. (e) The fiber diameters remained constant over the series. Scale bars are 10 μm.

As shown in Figure 1a–d, all of the fibers were smooth, straight, and bead-free. The diameter of all different PCL-N_3_ fibers was in a similar range from 800 to 1200 nm without significant differences between each group (Figure 1e). After the spinning process, we wanted to confirm if the azide functionalities survived the process. Using ^1^H NMR spectroscopy, we were able to confirm that the azide-functionalized end groups (CH_2_–N_3_ at 3.3 ppm) survived the spinning process (10% PCL-N_3_ scaffolds in CDCl_3,_Figure S2). Furthermore, FTIR analysis of the as-spun scaffolds (using an ATR stage) showed the characteristic azide peak was clearly present in the 10% PCL-N_3_ scaffolds. Although a low signal, this absorption intensity is as expected based on the the intesity of the azide in the pristine PCL-N_3_ (Figure S3, expected 1.6%, observed 1% absorbance).

### Surface Click of PCL-N_3_-Electrospun Fibers with Alkyne Megastokes

**Figure 2:**
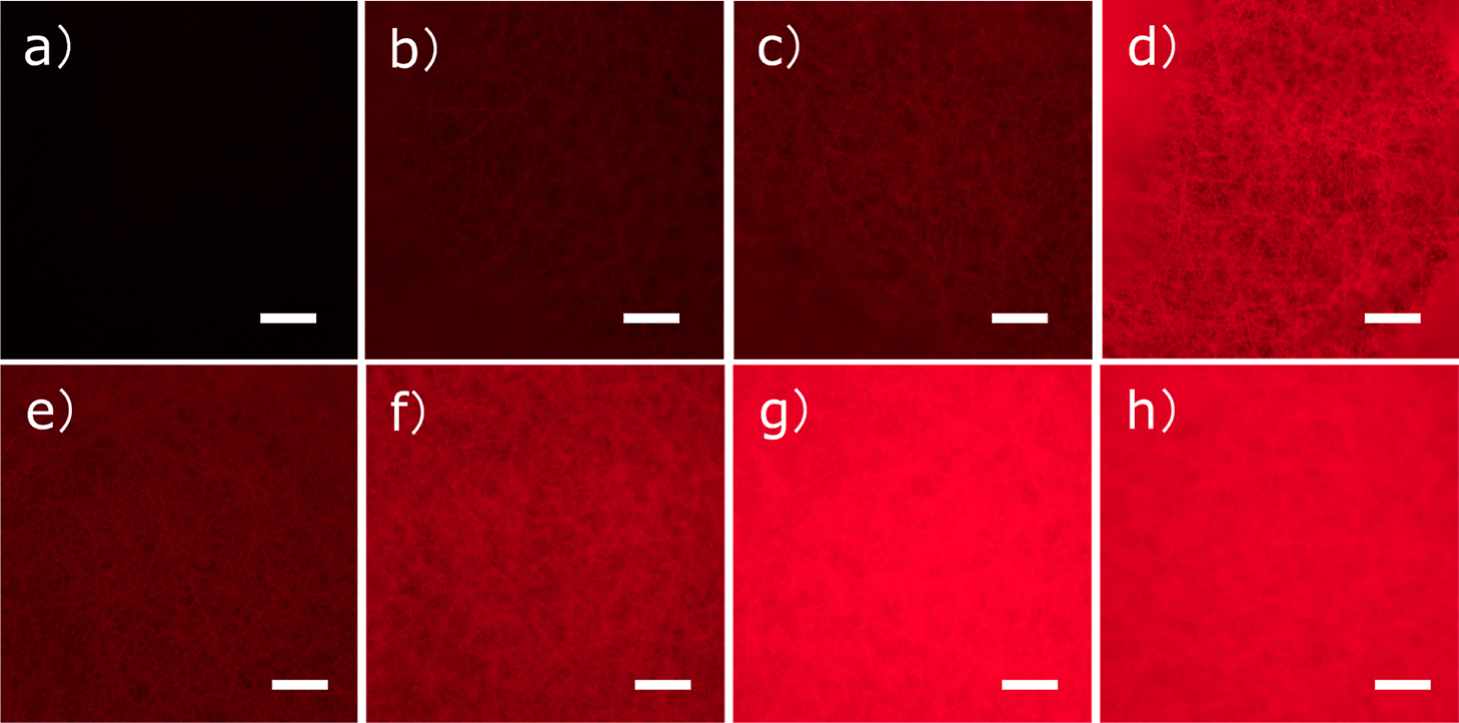
Fluorescence images of the different PCL-N_3_ fiber scaffolds containing: (a) 0, (b) 1, (c) 5 and (d) 10% PCL-N_3_ labeled with Alkyne Megastokes after 1 h. A time-course study (e–h) showing the effect of the click reaction time on the fluorescence intensity of the 5% PCL-N_3_ fibers (e: 15 min; f: 30 min; g: 2 h and h: 4 h). The fluorescence of MegaStokes was imaged using the excitation/emission 587/610 nm filters. Scale bars are 100 μm.

To confirm the availability of azide groups on the fiber surface and to optimize the functionalization procedure, we ran a model reaction with a fluorescent probe. We utilized Alkyne MegaStokes 673, an alkyne-containing fluorescent dye, in order to test and optimize the copper (I)-catalyzed azide–alkyne cycloaddition (CuAAC) reaction. Herein, we varied both the amount of azide spun into the samples and the time of reaction for the CuAAC coupling. Immediately apparent, fluorescence images of the fibrous scaffolds confirmed that the alkyne dye reacted with the azide groups on the surface (Figure 2a–d). Virtually no dye signal was observed in a control experiment carried out using the 0% PCL-N_3_ (Figure 2a), and an increase in the overall fluorescence intensity of the PCL-N_3_ fibers with an increasing percentage of PCL-N_3_ was observed in the fluorescence images (Figure 2a–d). Quantification of the fluorescence intensity indicated that there was a significant difference between 10% PCL-N_3_ electrospun scaffolds and the other electrospun scaffolds, where the 10% PCL-N_3_-electrospun scaffolds showed the highest fluorescence intensity compared to the others (***p < 0.001) (Figure S4a). The fluorescent labeling on the PCL-N_3_-electrospun scaffolds confirmed that the amount of a model small molecule could be easily controlled by changing the concentration of PCL-N_3_ in the scaffolds. Moreover, the effect of the reaction time on the click reaction between the 5% PCL-N_3_ fibrous scaffolds and Alkyne MegaStokes was investigated at 15 min, 30 min, 2 h and 4 h. As shown in Figure 2e–h, with the increase in the reaction time, the fluorescence intensity increased gradually at first and then reached a maximum fluorescence after 2 h. This finding was also confirmed by fluorescence quantification data, as shown in Figure S4b.

### Functionalization of Scaffolds with Alkynyl-NTA

Using the optimized functionalization procedure, NTA was then conjugated to the scaffold library with varying amounts of PCL-N_3_. The functionalization procedure was a straightforward CuAAc reaction at room temperature overnight, highlighting the mildness of the approach. After removal of the residual copper *via* EDTA chelation and washing, the functionalized scaffolds were investigated for the fidelity of the fibers and the presence of NTA.

**Figure 3:**
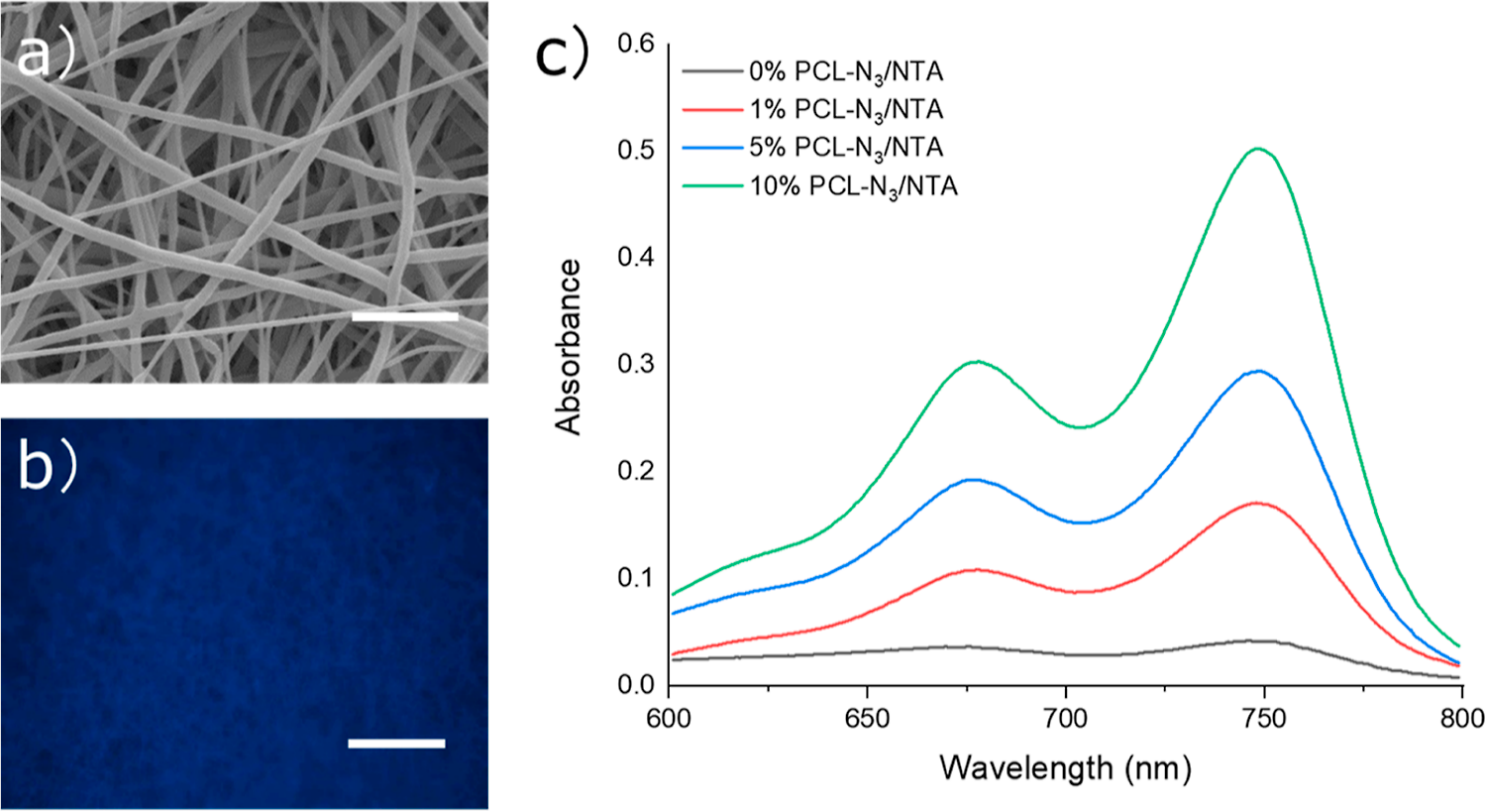
Characterization of the electrospun scaffolds functionalized with NTA. (a) SEM images of the 10% PCL-N_3_/NTA scaffolds showed no major changes in the fiber morphology (complete SEM images in Figure S5). (b) Soaking the scaffolds in a solution of 6-aminofluorescein labeled only the NTA functionalized scaffolds, supporting the successful coupling of NTA to the fibers (complete images in Figure S7). (c) The methylene blue assay showed that the scaffolds released H_2_S in a concentration-dependent manner *via* control of the PCL/PCL-N_3_ polymer feedstock ratio during spinning. Scale bars in (a,b) are 10 and 100 μm, respectively.

SEM images (Figures 3a and S5) of the different PCL-N_3_ fibrous scaffolds after the click reaction showed that the reaction conditions and coupling did not substantially affect the fiber morphology. The fibers were still uniform and smooth, and no significant morphological changes were observed from the SEM images.

Again, using FTIR, we observed the disappearance of the characteristic azide vibration band postfunctionalization (Figure S6), confirming the consumption of the azide in the reaction. In order to more directly probe the conjugation of NTA to the scaffold, we employed a qualitative fluorescence experiment. Alkynyl-NTA contains a thioanhydride; thus, the ring-opening of the NTA can be readily effected with an amine (also the initial step in the proposed mechanism for H_2_S release), resulting in the formation of an amide bond. Correspondingly, a 6-aminofluorescein dye was utilized to detect the immobilization of NTA on the 10% PCL-N_3_ scaffolds. Both the NTA functionalized (10% PCL-N_3_/NTA) and the nonfunctionalized (10% PCL-N_3_) fibrous scaffolds were immersed into an ethanol solution of 6-aminofluorescein. Fluorescent microscopy images showed no evident fluorescence on the nonfunctionalized scaffolds (Figure S7), yet a strong fluorescence on the NTA-functionalized scaffolds (Figure 3b). Quantification of the fluorescence intensity also confirmed that there was a significant difference between the 10% PCL-N_3_/NTA-electrospun scaffolds and the 10% PCL-N_3_-electrospun scaffolds (*p* < 0.001) (Figure S7c). This result more directly suggested successful click conjugation of the alkynyl-NTA to the PCL-N_3_ fibers.

### Quantification of H_2_S Release

After good evidence for the conjugation of NTA to the surface of the scaffolds, we next investigated the release of H_2_S from the NTA. In this two-stage process, the NTA must be opened by a nucleophile such as glycine to release COS; then, the COS must be converted into H_2_S by CA. Furthermore, we also aimed to investigate the ability of azide level incorporation to translate into a controlled release of H_2_S. We utilized the methylene blue assay to quantify the amount of H_2_S released.^47^ Briefly, after treatment of the NTA with glycine in PBS, COS was converted into H_2_S by CA. This H_2_S can then be quantified spectrophotometrically *via* the generation of methylene blue. As shown in Figure 3c, the amount of H_2_S detected increased with increasing amounts of PCL-N_3_ contained in the scaffold. Based on a calibration curve using Na_2_S (Figure S8), we determined that at 60 min, the concentration of H_2_S in solution was approximately 4.1, 7.0, and 12 nmol of H_2_S/mg of scaffold for the 1, 5, and 10% PCL-N_3_/NTA scaffolds, respectively. Investigating the kinetics of release in the methylene blue assay, we observed that the H_2_S concentration peaked quickly and remained steady from about 15–45 min (Figure S9). Conveniently, the amount of H_2_S delivered can be simply tuned by changing the amount of PCL-N_3_ in the fabricated scaffold. The steady release of H_2_S over 45 min under the accelerated conditions of the methylene blue assay indicates the potential for a slower release under *in vitro*/*vivo* conditions.

### Cellular Behaviors of HUVECs on Different PCL-N_3_/NTA Fibrous Scaffolds

To evaluate the biocompatibility of the NTA-functionalized scaffolds, we initially tested the viability of HUVECs cultured on the scaffolds for 1 day using alive/dead staining. As shown in Figure S10, nearly all the cells remained viable (green staining), and very few dead cells (red staining) were detected. The live cells attached to the surface of the scaffolds and started spreading on day 1. The percentage of living cells on 0% PCL-N_3_, 1% PCL-N_3_/NTA, 5% PCL-N_3_/NTA, and 10% PCL-N_3_/NTA scaffolds were quantified at 96 ± 2, 97 ± 4, 96 ± 2, and 98 ± 1%, respectively. These short-term results suggested that the NTA-functionalized electrospun fibrous scaffolds are noncytotoxic, suitable for cell culture, and that the NTA functionalization process does not significantly affect cell attachment or release cytotoxic amounts of H_2_S.

With the confirmation of short-term cytocompatibility, we then wanted to observe the effects of the dose-dependent release of H_2_S on the behavior of the HUVECs *in vitro*. The gasotransmitter is known to increase the proliferation of HUVECs, so our first efforts were to quantify this effect using the PrestoBlue metabolic activity, DNA content, and Ki67 proliferation assays. In these studies, we relied on endogenous CA to convert COS into H_2_S based on work from Pluth, Chakrapani, and Xian confirming this reaction *in vitro*.^48−50^

**Figure 4:**
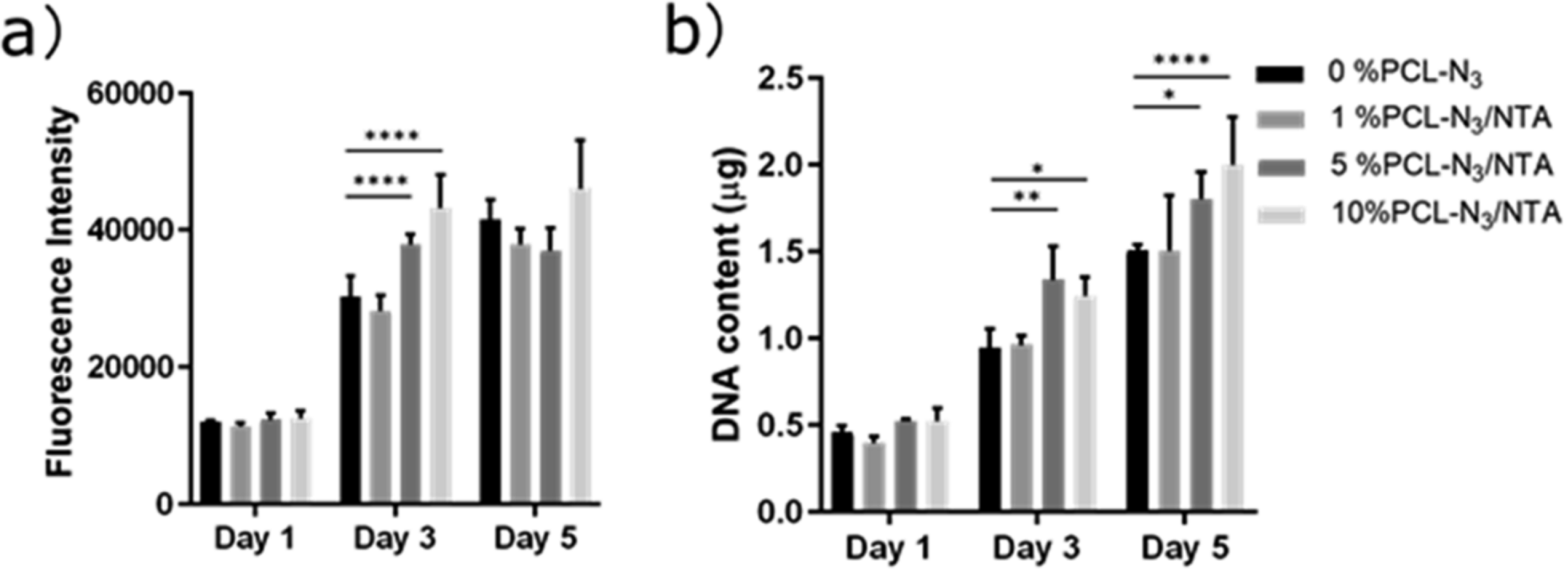
The viability (a) showed an initial increase and then a plateau during cell culture, while the proliferation (b) showed consistent increases of HUVECs cultured for 5 days on the different NTA functionalized scaffolds in EGM. *P ≤ 0.05, **P ≤ 0.005, ****P ≤ 0.0001.

The cell viability and metabolic activity of HUVECs at longer time points (1, 3, and 5 days) were evaluated with the PrestoBlue assay. We observed an increasing cell metabolic activity for all conditions over 5 days, although substantial differences between the different scaffolds were noticed already after 3 days of culture (Figure 4a). HUVECs cultured on the 5% PCL-N_3_/NTA and 10 %PCL-N_3_/NTA fibrous scaffolds showed a statistically significant increase in metabolic activity on day 3 compared to the cells on the 0% PCL-N_3_ and 1% PCL-N_3_/NTA scaffolds (*P* < 0.0001). There was no significant difference observed on day 5, suggesting a more pronounced effect of H_2_S release on the shorter timescales of the experiment. The higher metabolic activity of the cells on the 5% PCL-N_3_/NTA and 10% PCL-N_3_/NTA fibrous scaffolds on day 3 suggests that the higher NTA functionalization has positive effects on the viability of endothelial cells.

In order to further gain insights into the effects of H_2_S release on the behavior of HUVECs, we next investigated cell proliferation *via* total the DNA content on days 1, 3, and 5 (Figure 4b). The DNA quantification on day 3 had a similar trend as presented in the metabolic activity on day 3, showing a statistically significant increase in the proliferation on the 5% PCL-N_3_/NTA- and 10% PCL-N_3_/NTA-functionalized scaffolds compared to the 0% PCL-N_3_ and 1% PCL-N_3_/NTA scaffolds. Furthermore, a significant increase in cell proliferation on the 5% PCL-N_3_/NTA and 10% PCL-N_3_/NTA scaffolds was also observed on day 5.

**Figure 5:**
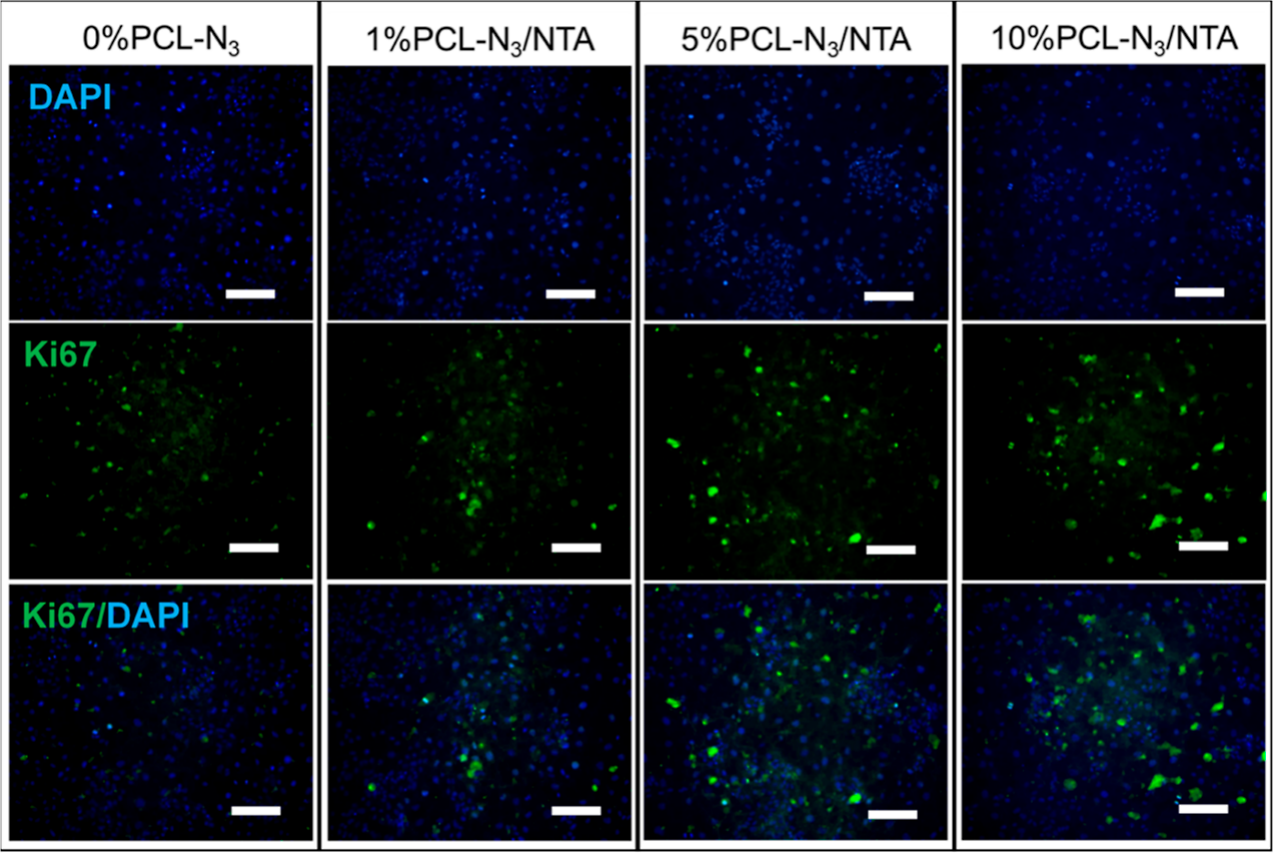
Cell proliferation was visualized by immunofluorescent staining using the anti-Ki67 antibody in HUVECs cultured on the different fibrous scaffolds after 5 days. (top) DAPI/cell nuclei, (middle) Ki67/proliferative cells, (bottom) merge. Scale bars are 100 μm.

Next, Ki67 staining was performed to identify cells actively in the proliferation and growth phases of the cell cycle. As shown in Figure 5, on day 5, the intensity of Ki67 fluorescence-positive cells in the 5% PCL-N_3_/NTA and 10% PCL-N_3_/NTA fibrous scaffolds was higher than that in the 0% PCL-N_3_ and 1% PCL-N_3_/NTA scaffolds. These results suggested that even after 5 days, there remained an increase in the proportion of actively proliferating cells in the functionalized scaffolds with a greater NTA content (see all quantitative data in Figure S11).

Interestingly, throughout the experiments, the 1% functionalized scaffolds showed no difference from the control, while the viability (day 3) and proliferation (day 3 and 5) on the 5 and 10% scaffolds increased in a dose-dependent manner. Previous studies showed that exposure of endothelial cells to H_2_S (60 μM) led to a higher cell proliferation, while a lower concentration of 6 μM H_2_S showed no significant effect on the increase in the cell number,^30^ in line with our findings. In order to test the generality of the observed dose-dependent effect, cell viability and proliferation was also tested on FBS-coated scaffolds (Figure S12). Despite problems with cell adhesion and detachment, we could also observe the higher viability and proliferation of HUVECs on the H_2_S functionalized scaffolds over a 5 day period. Taken together, the above results show that all fibers acted as a suitable scaffold for cell attachment and proliferation, and the activity of the NTA as an H_2_S donor resulted in faster cell proliferation in a dose-dependent manner.

### Morphological Analysis and Immunostaining of HUVECs Grown on Different PCL-N_3_ Fibrous Scaffolds

**Figure 6:**
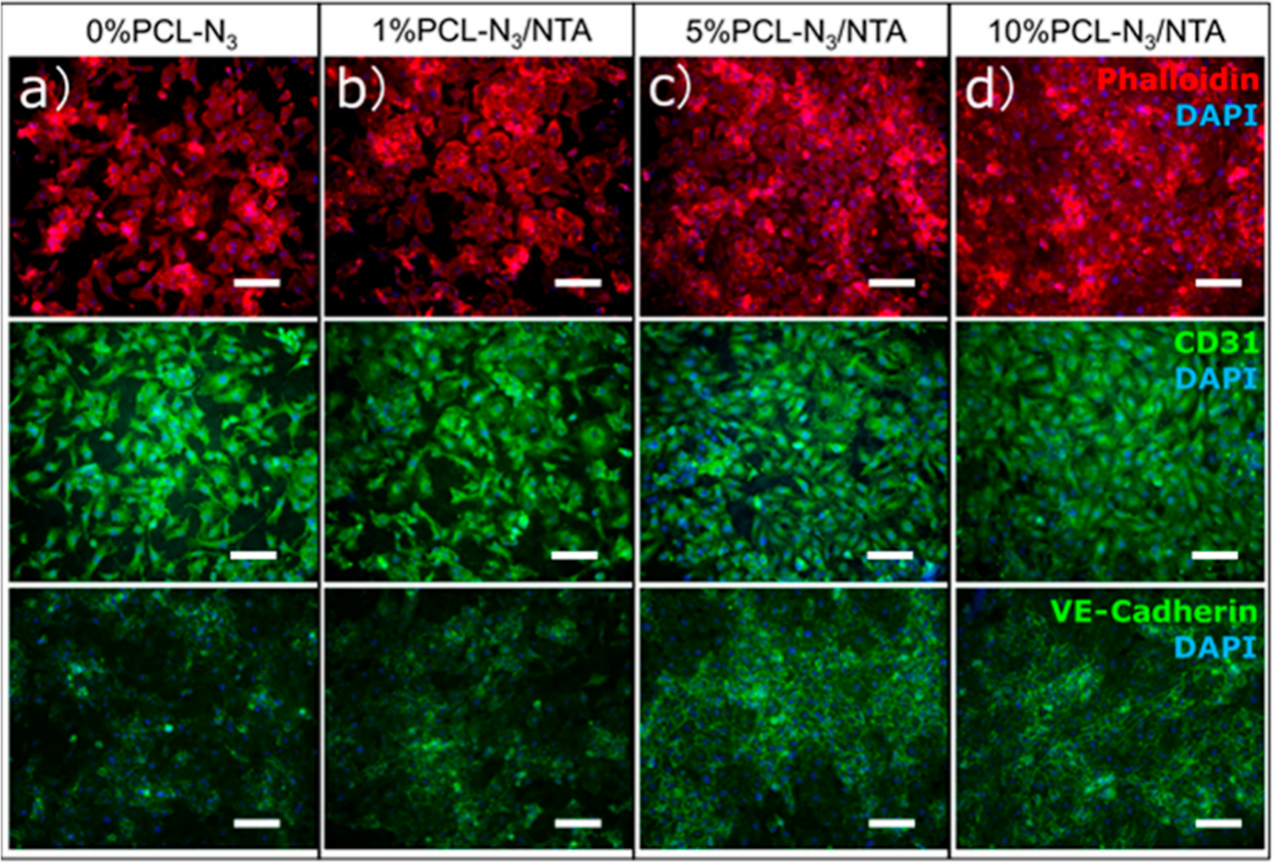
The immunofluorescence staining images showing the nuclei (blue) and the expression of F-actin (red), CD31 (green), and VE-Cadherin (green) of HUVECs grown on the (a) 0% PCL-N_3_ fibrous scaffolds, (b) 1% PCL-N_3_/NTA fibrous scaffolds, (c) 5% PCL-N_3_/NTA fibrous scaffolds, and (d) 10% PCL-N_3_/NTA fibrous scaffolds after 5 days. Scale bars are 100 μm.

Knowing that the H_2_S-releasing scaffolds increased proliferation, we then wanted to investigate if there were morphological or phenotypic differences induced by this H_2_S signaling. Correspondingly, HUVEC behavior, cytoskeleton development, and intercellular junctions were investigated on the different PCL-N_3_ scaffolds by fluorescent staining after 5 days of culture. To observe the cellular morphology, we used phalloidin and DAPI to stain the cell cytoskeletons and nuclei, as shown in Figure 6. HUVECs adhered well to all the fibrous scaffolds, and distinct cytoskeletal structures were observed. The HUVECs grown on the 5% PCL-N_3_/NTA and 10% PCL-N_3_/NTA fibrous scaffolds rapidly proliferated and showed more confluence in comparison to the HUVECs cultured on the 0% PCL-N_3_ and 1% PCL-N_3_/NTA scaffolds, as reflected in the viability and proliferation studies above. To confirm the morphology and spreading of HUVECs on the different scaffolds, SEM was also performed after 5 days in culture (Figure S13). Interestingly, we observed that the HUVECs on the 10% PCL-N_3_/NTA fibrous scaffolds showed numerous aggregated dots on their cell membranes. We hypothesize that the dotted pattern produced by these HUVECs can be correlated to von Willebrand factor (vWF) dots, as previously reported.^51^

In order to study the functional development of HUVECs on the fibers, we analyzed the expression of the universal endothelial cell markers: CD31, VE-Cadherin, and vWF. CD31 staining (Figure 6, middle), widely recognized as an endothelial cell marker, suggested that HUVECs cultured on the different scaffolds maintained their endothelial cell phenotype. The intercellular junction of HUVECs, that is, the basic adhesion junction in endothelial cells, on the scaffolds was further revealed by VE-Cadherin staining (Figure 6, bottom). HUVECs on the 0% PCL-N_3_ and 1% PCL-N_3_/NTA scaffolds were mainly isolated (low VE-cadherin staining), while the 5% PCL-N_3_/NTA and 10% PCL-N_3_/NTA fibrous scaffolds showed a nearly confluent cell monolayer with an increased staining of tight junctions. The firm intercellular junctions were formed on the 5% PCL-N_3_/NTA and 10% PCL-N_3_/NTA fibrous scaffolds, which would be beneficial for generating a stabilized confluent endothelial monolayer and maintaining the integrity of an endothelium. A small, dotted pattern of vWF was clearly observed in Figure S14, which indicated some of the HUVECs could produce vWF within the cytoplasm when cultured on the different PCL-N_3_/NTA fibrous scaffolds and supports the observation of vWF on the cellular surface in the SEM images (Figure S13). While vWF has a complex role in the regulation of angiogenesis, its production has been shown to both inhibit vascular network formation^52^ and induce vessel maturation,^53^ suggesting that these cells could be signaling a move toward maturation.

### *In Ovo* CAM Assay

**Figure 7:**
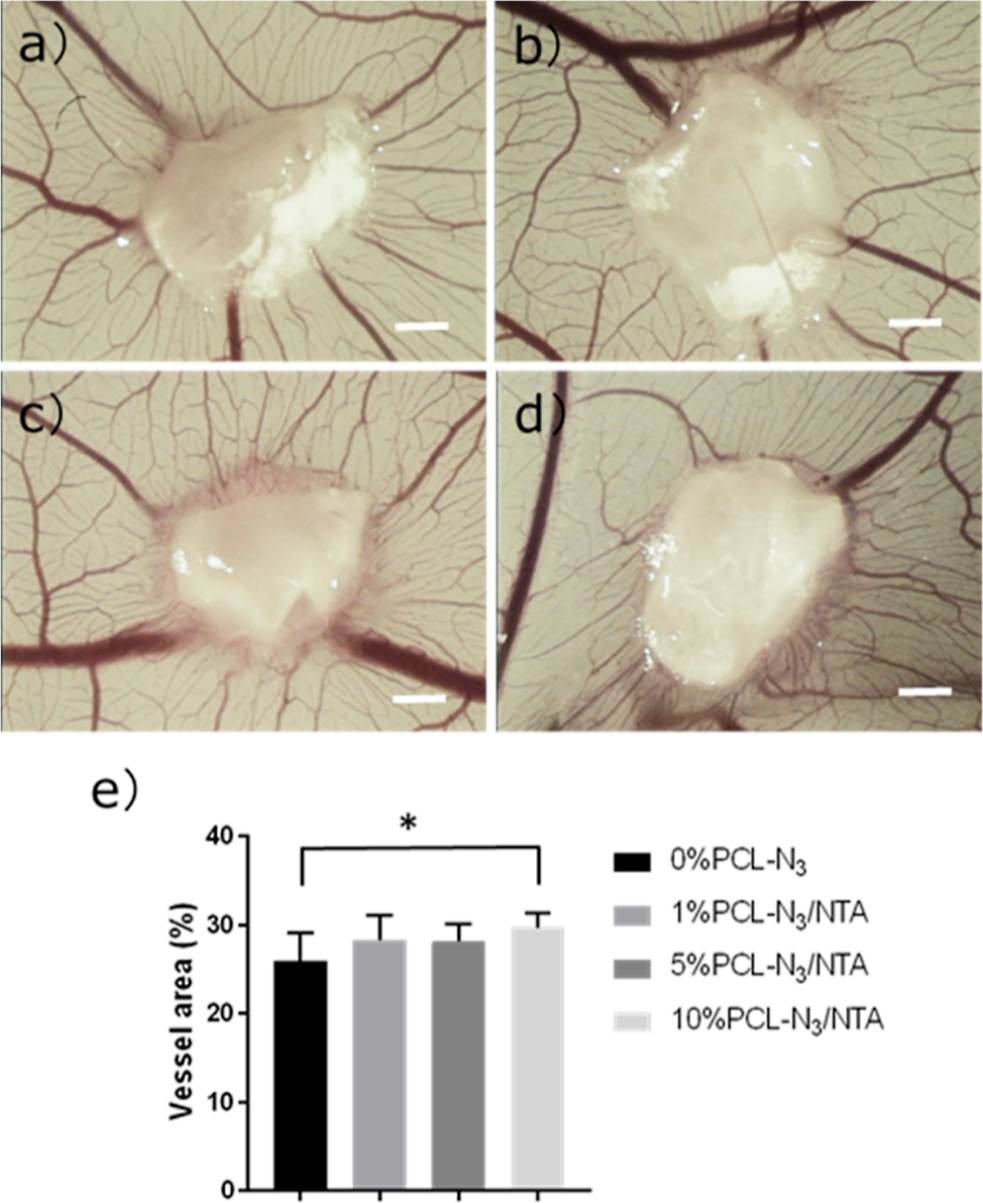
The effects of the different NTA-functionalized scaffolds on angiogenesis using a CAM assay. Shown are representative images of the capillary vessel growth surrounding the (a) 0% PCL-N_3_, (b) 1% PCL-N_3_/NTA, (c) 5% PCL-N_3_/NTA, and (d) 10% PCL-N_3_/NTA fibrous scaffolds after 4 days of implantation. (e) The quantification of the vessel area around different fibrous scaffolds. Scale bars are 1 mm. *P ≤ 0.05.

While promising results came from these scaffolds in the highly controlled *in vitro* studies with HUVECs, we wanted to know whether our approach was effective at stimulating angiogenesis in a complex *in ovo* environment. We turned to the CAM assay to assess whether the NTA-functionalized scaffolds could promote angiogenesis. Figure 7a–d shows the images of blood vessels around 0% PCL-N_3_, 1% PCL-N_3_/NTA, 5% PCL-N_3_/NTA, and 10% PCL-N_3_/NTA scaffolds after 4 days of incubation on the egg membrane. Both the 5% PCL-N_3_/NTA and 10% PCL-N_3_/NTA scaffolds implanted on chicken embryo CAM presented a visual increase of vessels surrounding the scaffolds; the capillary vessels grew radially toward and away from the scaffolds.

To quantify the ability of NTA-functionalized scaffolds to induce angiogenesis, the blood vessel area was determined using automated image processing (Figure 7e, processed images shown in Figure S15). The quantitative results indicated that the blood vessel area surrounding the 10% PCL-N_3_/NTA scaffolds (29.8 ± 1.7%) was significantly higher than that of the 0% PCL-N_3_ scaffolds (25.9 ± 3.2%) (*P* = 0.0148). This means that implantation with 10% PCL-N_3_/NTA scaffolds led to 1.15 times the vessel area (about a 15.4% increase) as compared to that on the control scaffolds. The CAM assay shows the potential of combining a tailorable scaffold with a powerful gasotransmitter for angiogenesis. The controllable functionalization of our scaffold allowed us to observe a dose-dependent response *in vitro* and to observe the necessity for higher amounts of H_2_S release when moving to *in vivo* applications. While several of the scaffolds were promising in cell culture, a high NTA loading of the scaffolds (10%) was needed to induce angiogenesis *in ovo*.

## Conclusions

We set out to create a simple and synthetic proangiogenic scaffold by incorporating both the physical and chemical components of the native ECM. By combining fibrous electrospun meshes (PCL, physical) with a novel H_2_S-releasing small molecule (NTA, chemical), we demonstrated that the addition of the NTA increased the angiogenic potential of the scaffolds. While the 5 and 10% functionalized scaffolds both showed promising *in vitro* results; ultimately, the 10% functionalized scaffold clearly showed the most angiogenic potential in the CAM assay. The H_2_S-releasing NTA scaffolds clearly provided a benefit, *in vitro* and *in ovo*, without the need for growth factors. While we attribute the proangiogenic effects to H_2_S, we cannot rule out some contribution from COS in these NTA-based donor systems. Future efforts focused on enzyme-triggered H_2_S donors may enhance the capabilities of this system by allowing cells themselves to trigger H_2_S release.

Furthermore, the controlled mixing strategy employed allowed a straightforward and high-fidelity postfabrication functionalization of the scaffold. We have demonstrated that alkynyl-NTA could be attached into PCL-N_3_-electrospun scaffolds *via* the CuAAC click reaction without impairing the fiber morphology of the scaffolds, and tunable amounts of functionalization and H_2_S release could be achieved. Surprisingly, the efficiency of the azide-to-H_2_S transformation (azide input to the amount of H_2_S observed) is high for a multistep process including fabrication, functionalization, release, and detection. This simple strategy is likely not limited to NTA functionalization but is a more general method to enable the facile postfabrication surface functionalization of other bioactive electrospun scaffolds from peptides and DNA to small molecules.

Overall, the NTA-functionalized fibrous scaffolds are promising to promote endothelial cell proliferation and increase the vessel density, both critical steps for angiogenesis in tissue engineering. The results from this study suggest a potentially powerful effect from H_2_S; even with the release complete in the course of a handful of hours, we still observed proangiogenic effects over 5 days. As we work toward H_2_S-releasing scaffolds with longer release profiles, we expect to see even greater effects. The ability to control vascularization without the need for growth factors such as VEGF, which is limited by scalability and off-target effects, represents a novel and attractive strategy in tissue engineering. Our simple approach employs scalable chemical processes and techniques while still maintaining high levels of vascularization *in ovo*. Remaining questions include the comparative performance of novel H_2_S-releasing scaffolds to more widely studied growth factor releasing scaffolds and the performance of H_2_S releasing scaffolds in the generation of vascularized large tissue models.

## Supporting Information Available

Additional experimental details, materials, and methods, including ^1^H NMR of polymers, quantification of functional density and fluorescence images, kinetic release studies of H_2_S, and detailed SEM of the scaffolds with and without cells (PDF)
