# Supplementary material for: Electrospun Scaffolds Functionalized with a Hydrogen Sulfide Donor Stimulate Angiogenesis
Source: ACS Appl Mater Interfaces. 2022 Jun 17;14(25):28628–38. doi: 10.1021/acsami.2c06686 (PMC9247975; doi:10.1021/acsami.2c06686)
Supplement: Supplementary file 1 — am2c06686_si_001.pdf [file am2c06686_si_001.pdf]

Supporting Information

Electrospun Scaffolds Functionalized with a Hydrogen Sulfide Donor Stimulate Angiogenesis

Tianyu Yao[1,2], Teun van Nunen[1], Rebeca Rivero[1], Chadwick Powell[3], Ryan Carrazzone[3], Lilian Kessels[4], Paul Andrew Wieringa[1], Shahzad Hafeez[1], Tim G.A.M. Wolfs[4], Lorenzo Moroni[1*], John B. Matson[3*], Matthew B. Baker[1*]

[1]Complex Tissue Regeneration, MERLN Institute for Technology-Inspired Regenerative Medicine, Maastricht University, Universiteitssingel 40, 6229 ER, Maastricht, the Netherlands

[2]Shaanxi Key Laboratory of Degradable Biomedical Materials and Shaanxi R&D Center of Biomaterials and Fermentation Engineering, School of Chemical Engineering, Northwest University, Taibai North Road 229, Xi'an, Shaanxi, 710069, China

[3]Chemistry Department Macromolecules Innovation Institute, Virginia Tech, 1075 Life Science Circle, Blacksburg, VA 24061, United States of America

[4]Department of Pediatrics, Universiteitssingel 50, 6229 ER, Maastricht University, Maastricht, the Netherlands

*E-mail: l.moroni@maastrichtuniversity.nl; jbmatson@vt.edu; m.baker@maastrichtuniversity.nl

Table S1. Electrospinning parameters for PCL-80K and azide-functionalized fibers: 0%PCL-N_3, 1%PCL-N_3, 5%PCL-N_3 and 10%PCL-N_3.

Electrospun fibers	Total polymer (wt%)	PCL-80k :	PCL-2k :	PCL-N_3-2k	Voltage (kV)	Flow rate (mL/h)	Working Distance (cm)
0%PCL-N_3	15	90	10	0	23	1	20
1%PCL-N_3	15	90	9	1	20	1	20
5%PCL-N_3	15	90	5	5	20	1	20
10%PCL-N_3	15	90	0	10	20	1	20

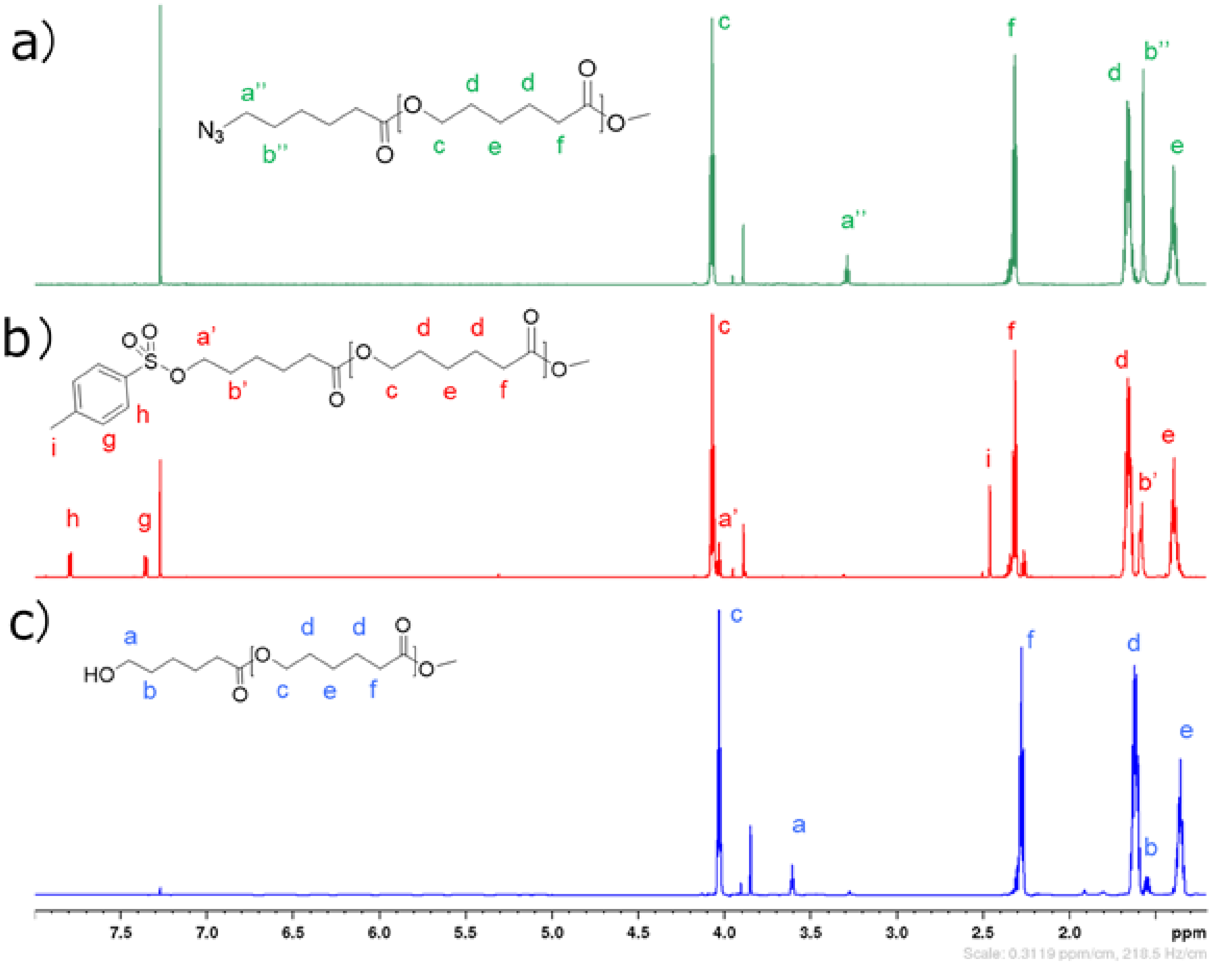

Figure S1. ^{1}H NMR (700 MHz) spectra of the (a) PCL-N_3, (b) PCL-OTS, and (c) PCL polymers in $CDCl_3$.

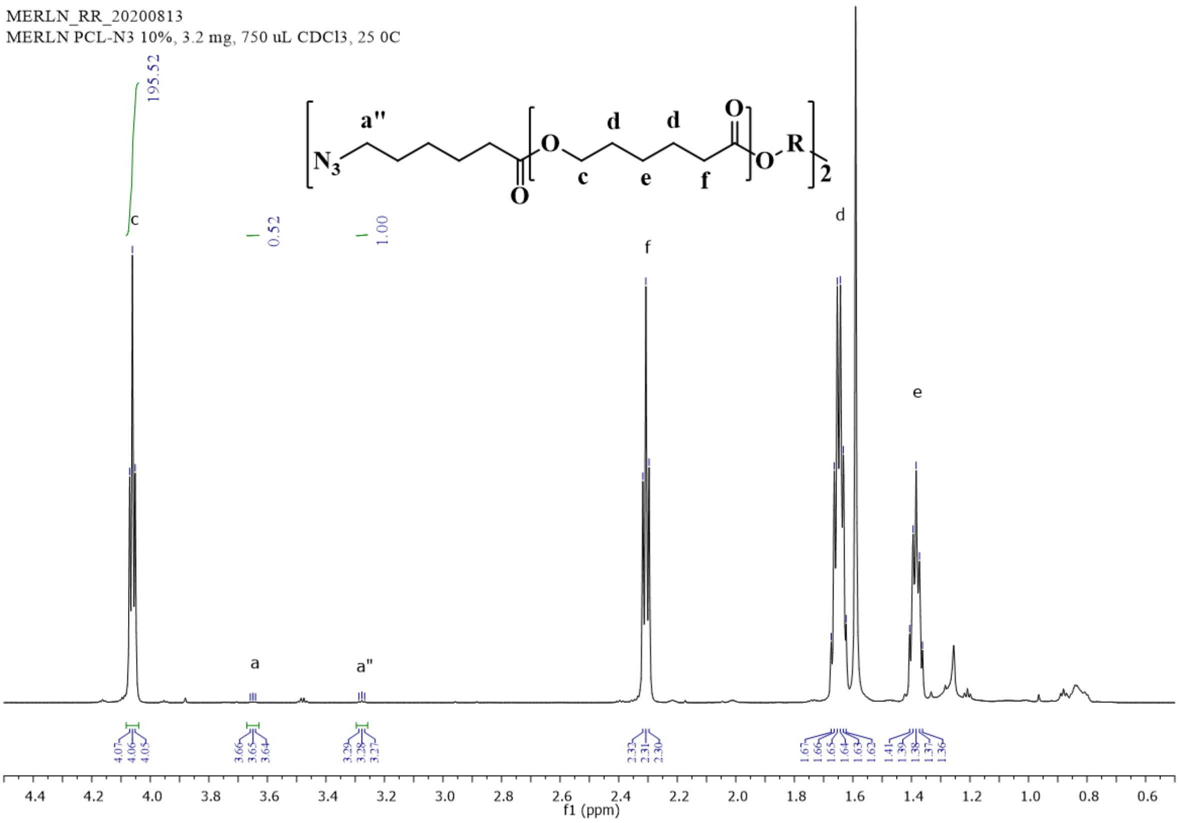

Figure S2. ^{1}H NMR (700 MHz) spectra of the electrospun scaffold containing 10% PCL-N_3, showing the fidelity of the azide post-fabrication. Peak labeled a is from the terminal alcohol of PCL (Figure S1), and represents the alpha methlyenes on the 80K PCL..

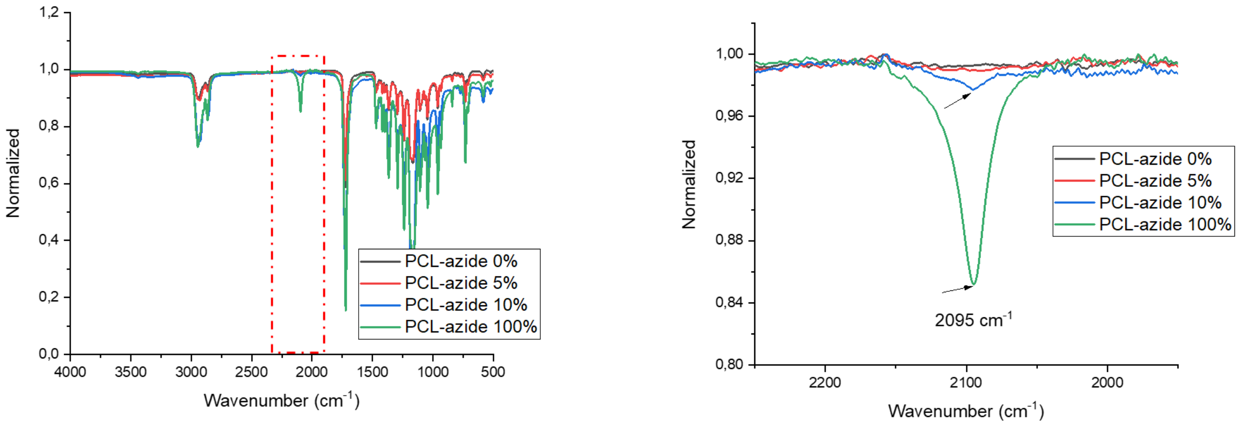

Figure S3. FTIR (ATR stage) showing the presence of the azide peak in the pure PCL-N_3 and in the PCL-N_3 10% scaffolds. The PCL-N_3 5% scaffolds were below the limit of detection.

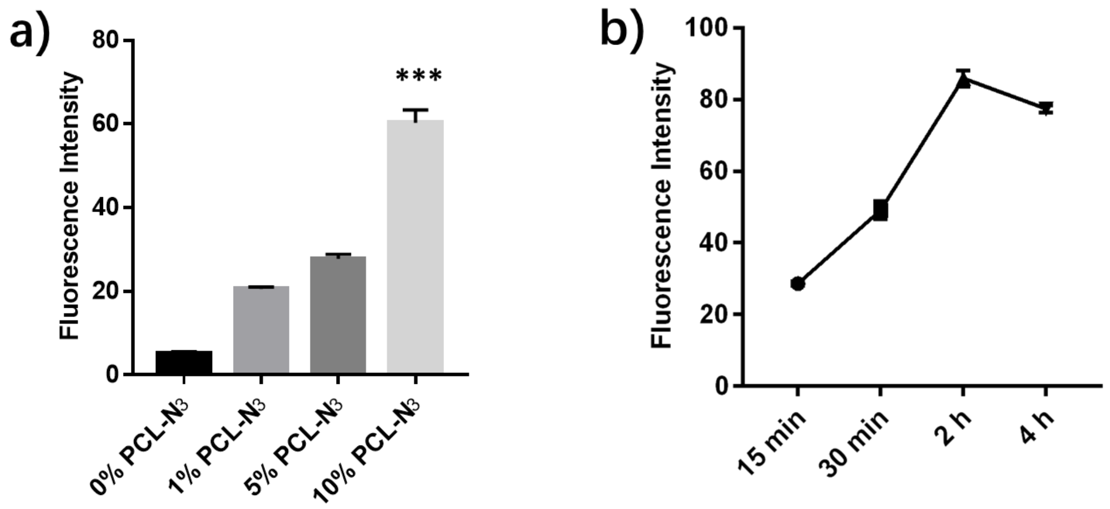

Figure S4. Quantification data of fluorescence intensity in Figure 2a-d. Figure 2a-d showed fluorescence images of different PCL-N_3 fibrous scaffolds after labelling with Alkyne Megastokes. (b) Quantification data of fluorescence intensity in Figure 2e-h. Figure 2e-h showed fluorescence images of 5% PCL-N_3 fibrous scaffolds reacted with Alkyne Megastokes at different times. ***$p \leq 0.001$.

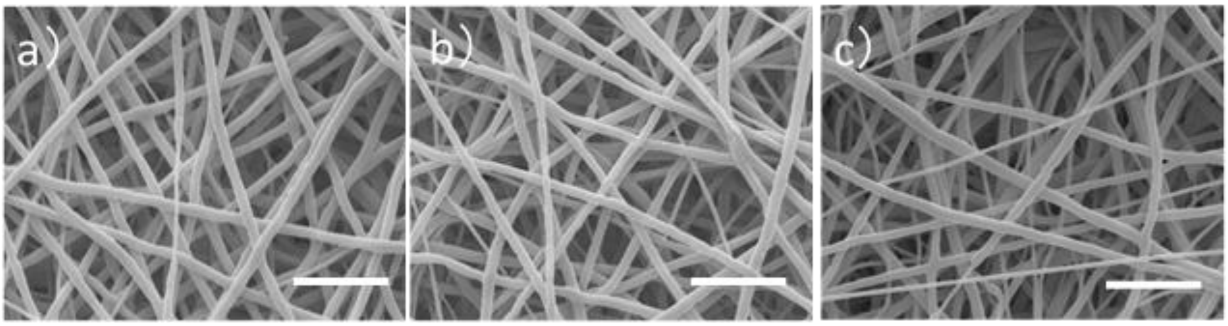

Figure S5. SEM images of (a) 1% PCL-N_3, (b) 5% PCL-N_3 and (c) 10% PCL-N_3 nanofibrous scaffolds after click reaction with alkynyl-NTA. Scale bars are 10 µm.

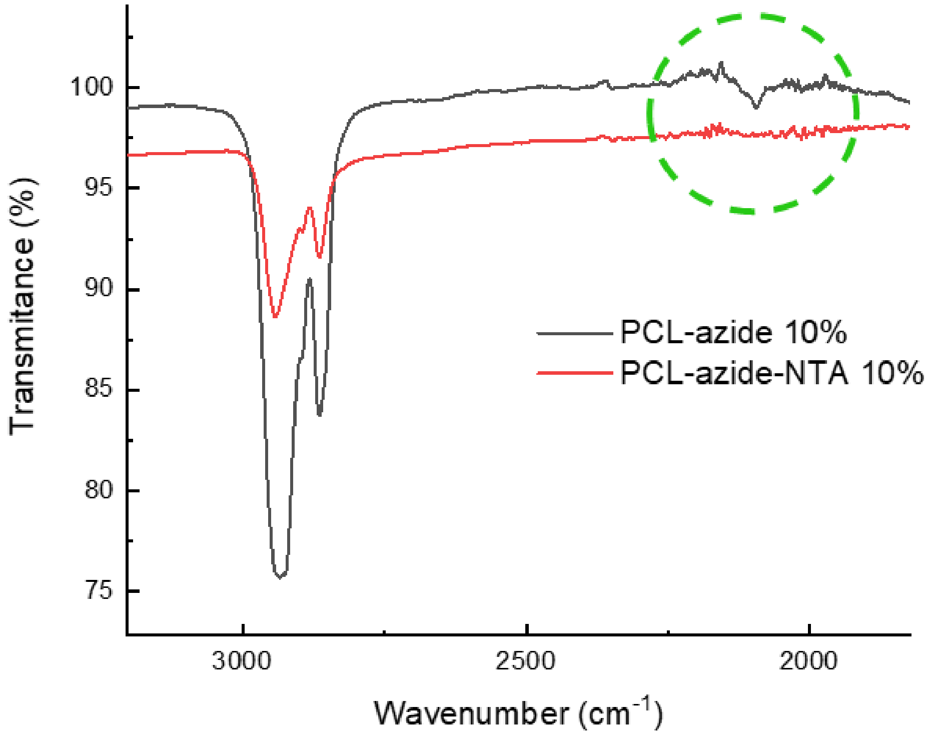

Figure S6. FTIR images of a) 10% PCL-N_3 electrospun scaffolds after immersion in the 6-Aminofluorescein solution, b) 10% PCL-N_3 electrospun scaffolds functionalized with alkynl-NTA and then labelled with 6-Aminofluorescein.

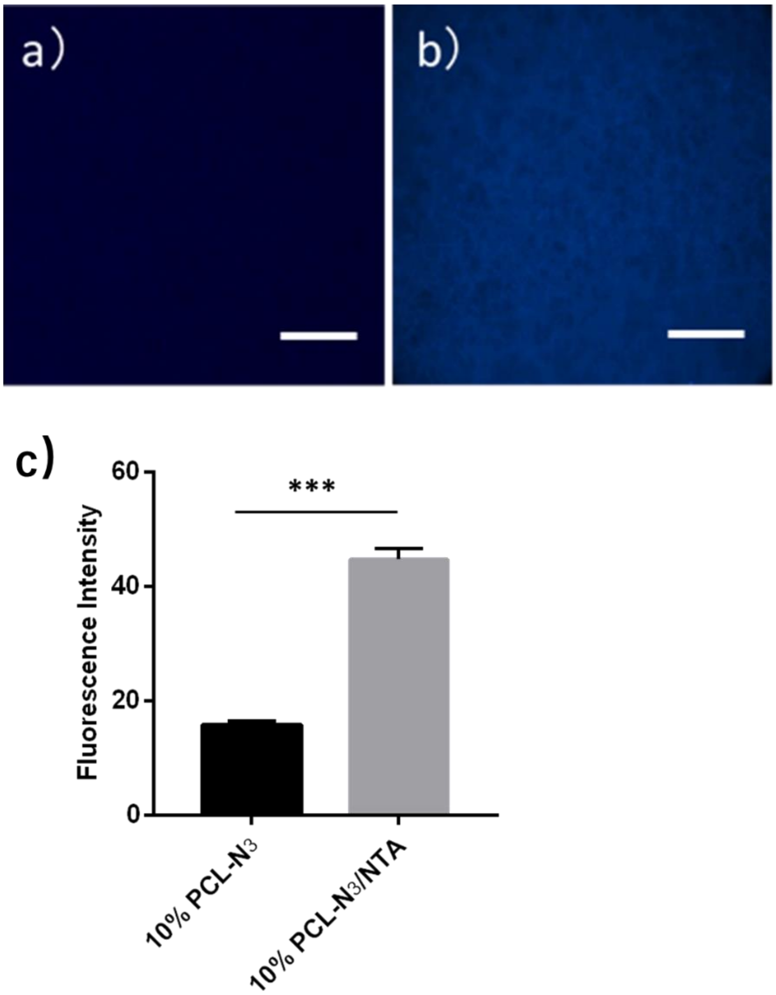

Figure S7. Fluorescence images of a) 10% PCL-N_3 electrospun scaffolds after immersion in the 6-aminofluorescein solution, b) 10% PCL-N_3 electrospun scaffolds functionalized with alkynl-NTA and then labelled with 6-Aminofluorescein. Scale bars are 100 µm. c) Quantification of fluorescence intensity in panels a and b. Scale bars are 100 µm. ***P $\leqslant$ 0.001.

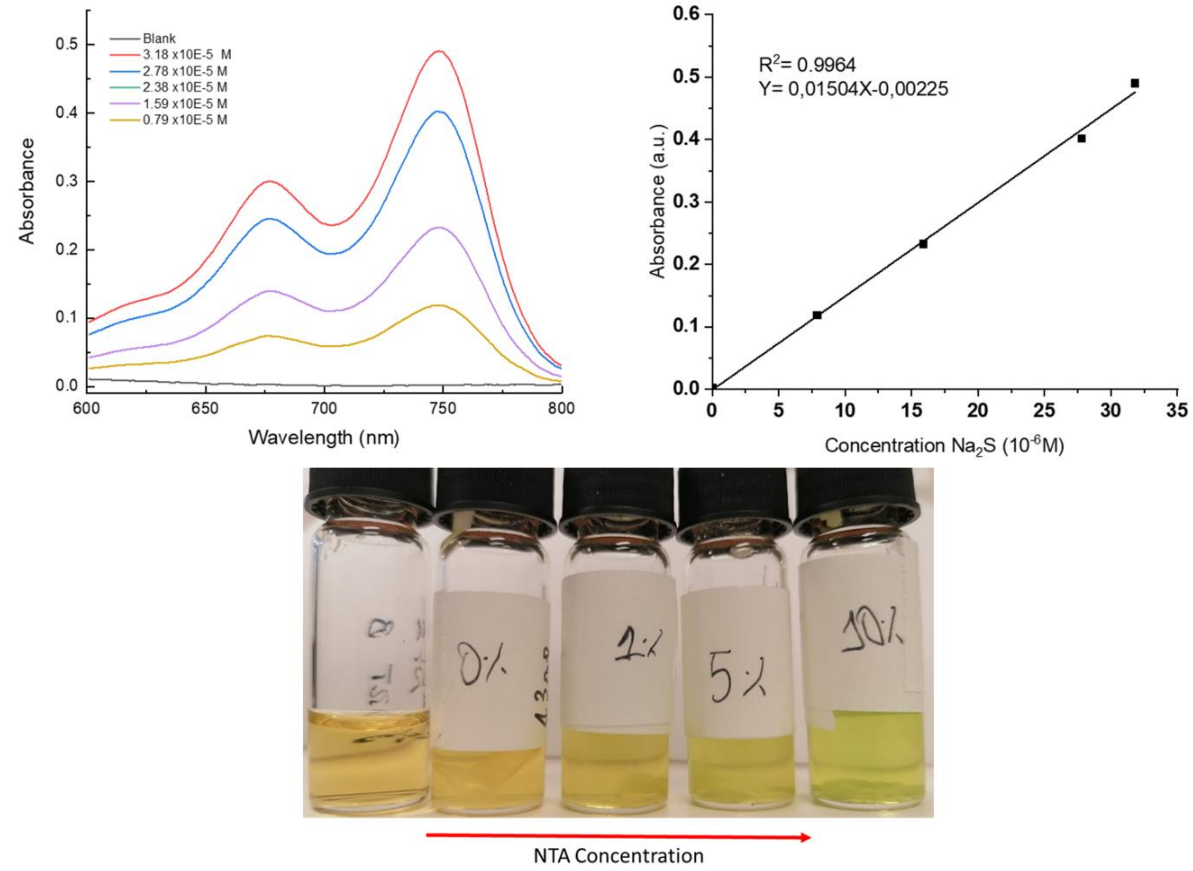

Figure S8. The (top left and top right) standard curve of Methylene Blue with Na_2S concentrations, (bottom) visual indications of methylene blue for the samples of blank, 0%, 1%, 5%, and 10% functionalized scaffolds (left to right) during the methylene blue assay.

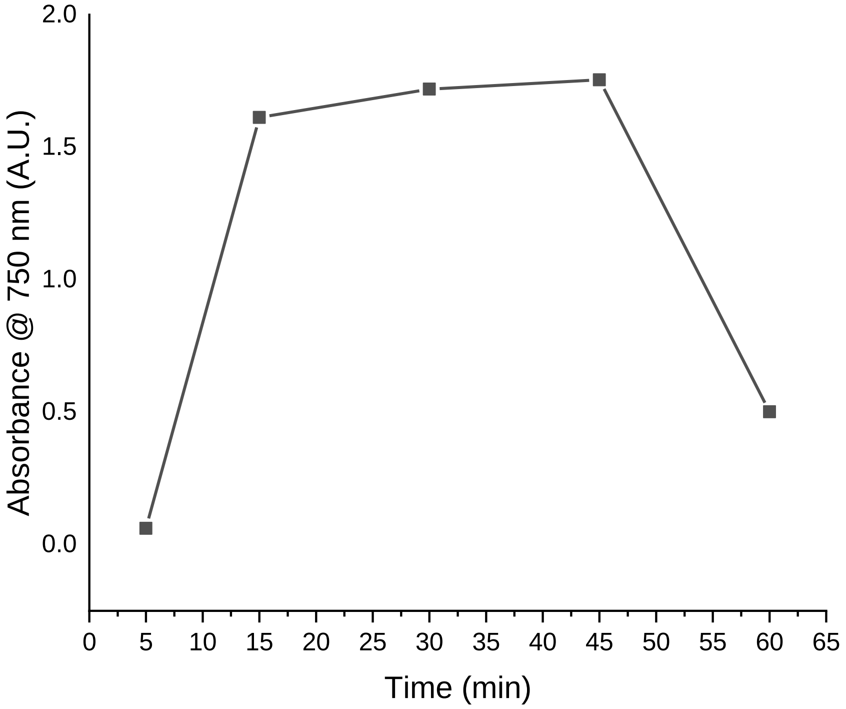

Figure S9. Time course measurements with the 10% functionalized scaffolds in the methylene blue assay showed a small induction period (5 min) with a rapid steady state release of H_2S over 15–45 minutes of the measurement.

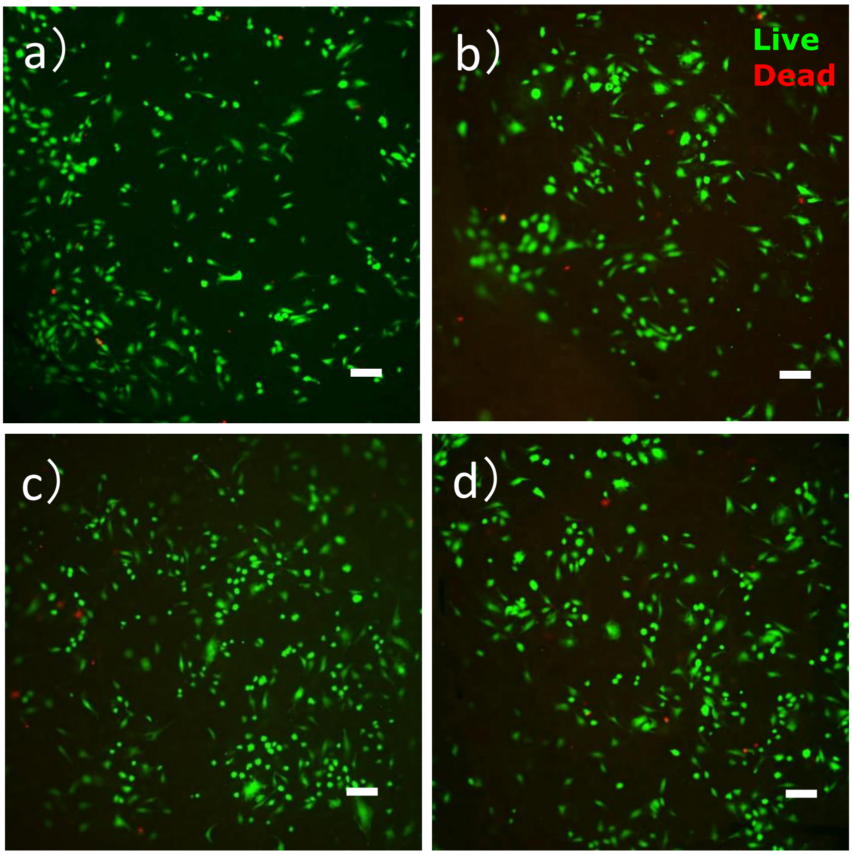

Figure S10. Live/dead staining images of HUVECs cultured on (a) 0%PCL-N_3 fibrous scaffolds, (b) 1%PCL-N_3/NTA fibrous scaffolds, (c) 5%PCL-N_3/NTA fibrous scaffolds and (d) 10%PCL-N_3/NTA fibrous scaffolds. Scale bars are 100 µm.

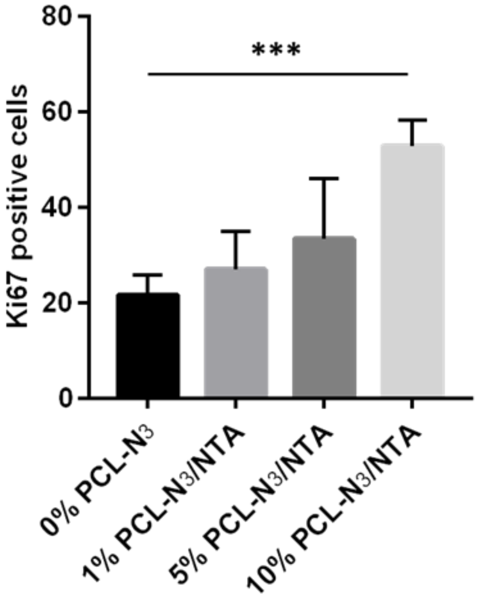

Figure S11. Quantification of Ki67 positive cells on different scaffolds. ***P ≤ 0.001.

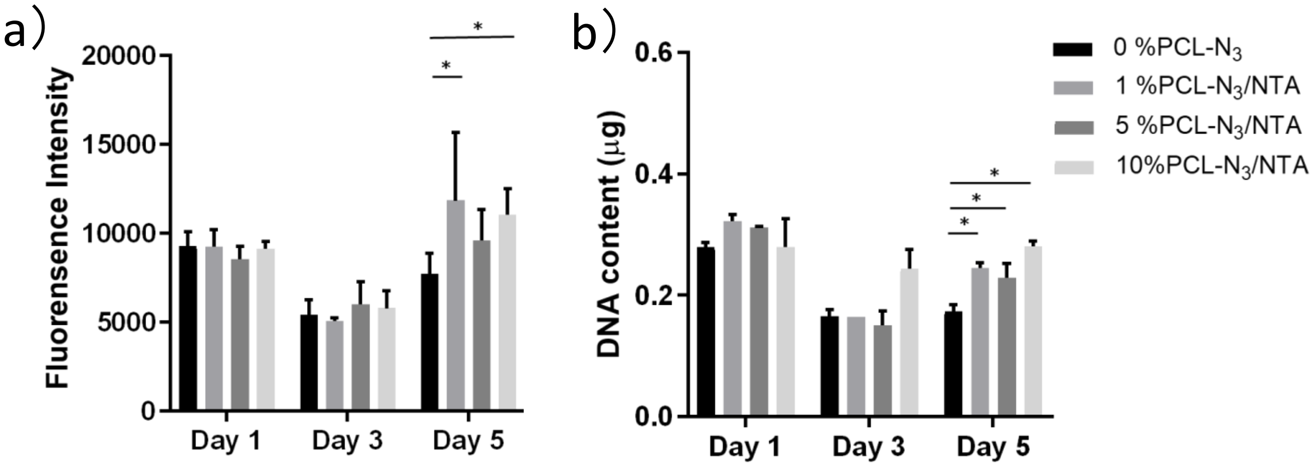

Figure S12. Viability (a) and proliferation via DNA quantification (b) of HUVECs cultured for 5 days on different NTA functionalized scaffolds (with FBS pre-coating). *P ≤ 0.05.

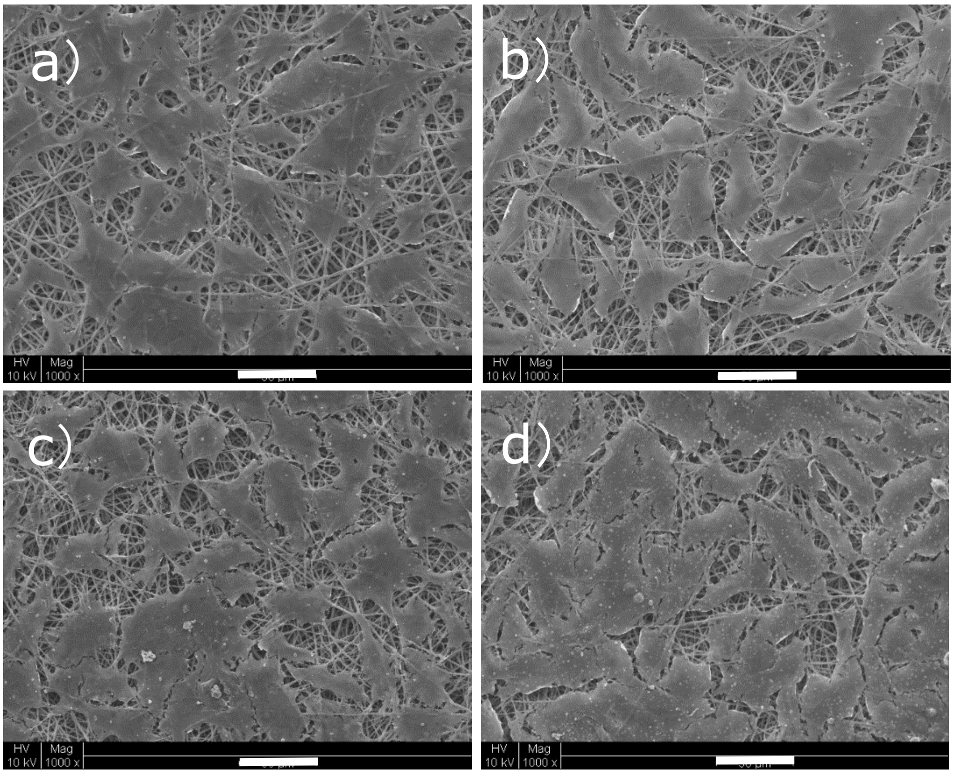

Figure S13. SEM images of HUVECs on (a) 0%PCL-N_3 fibrous scaffolds, (b) 1%PCL-N_3/NTA fibrous scaffolds, (c) 5%PCL-N_3/NTA fibrous scaffolds and (d) 10%PCL-N_3/NTA fibrous scaffolds after 5 days of culture. Scale bars are 50 µm.

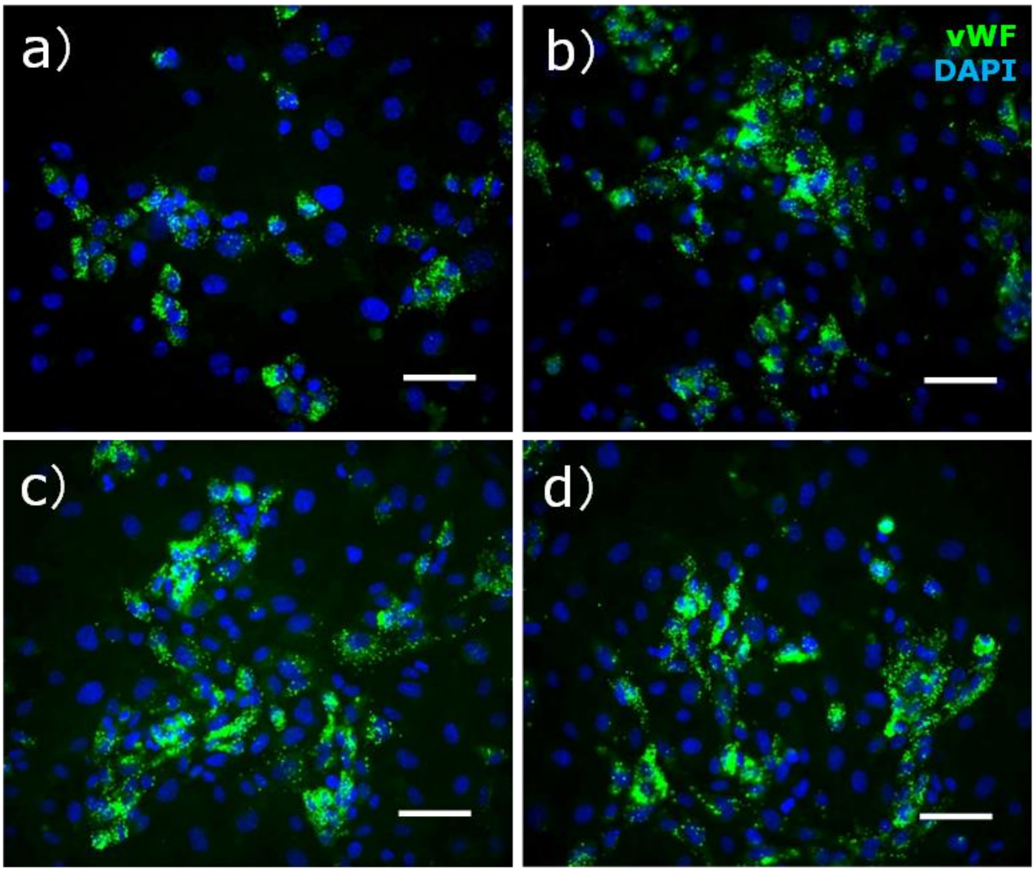

Figure S14. Expression of vWF on HUVECs grown on (a) 0%PCL-N_3 fibrous scaffolds, (b) 1%PCL-N_3/NTA fibrous scaffolds, (c) 5%PCL-N_3/NTA fibrous scaffolds, and (d) 10%PCL-N_3/NTA fibrous scaffolds after 5 days of culture. Scale bars are 50 µm.

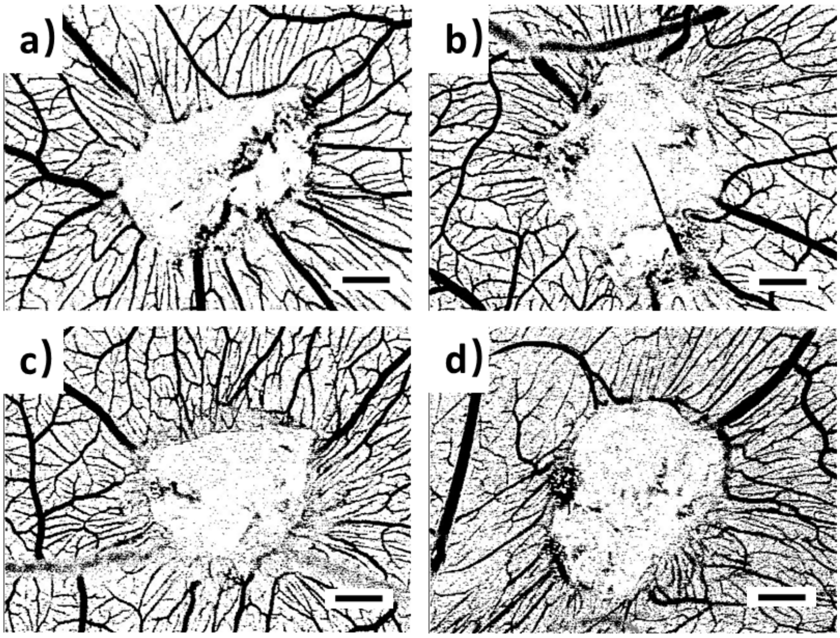

Figure S15. Processed images of (a) 0% PCL-N_3, (b) 1% PCL-N_3/NTA, (c) 5% PCL-N_3/NTA and (d) 10% PCL-N_3/NTA fibrous scaffolds implanted on the CAM after 4 days. Scale bars are 1mm.
